# CD45 inhibition in myeloid leukaemia cells sensitizes cellular responsiveness to chemotherapy

**DOI:** 10.1007/s00277-023-05520-y

**Published:** 2023-11-02

**Authors:** Maryam Ahmed S. Al Barashdi, Ahlam Ali, Mary Frances McMullin, Ken Mills

**Affiliations:** 1 Patrick G Johnston Centre for Cancer Research, Queen’s University Belfast, Belfast, Northern Ireland UK; 2 Haematology Department, C-Floor Tower Block, Belfast City Hospital, Belfast, Northern Ireland UK

**Keywords:** CD45, PTPRC, Common leukocyte antigen (CLA), AML, Myeloid leukaemia

## Abstract

Myeloid malignancies are a group of blood disorders characterized by the proliferation of one or more haematopoietic myeloid cell lineages, predominantly in the bone marrow, and are often caused by aberrant protein tyrosine kinase activity. The protein tyrosine phosphatase CD45 is a trans-membrane molecule expressed on all haemopoietic blood cells except that of platelets and red cells. CD45 regulates various cellular physiological processes including proliferation, apoptosis, and lymphocyte activation. However, its role in chemotherapy response is still unknown; therefore, the aim of this study was to investigate the role of CD45 in myeloid malignancies in terms of cellular growth, apoptosis, and response to chemotherapy. The expression of CD45 on myeloid leukaemia primary cells and cell lines was heterogeneous with HEL and OCI-AML3 cells showing the highest level. Inhibition of CD45 resulted in increased cellular sensitivity to cytarabine and ruxolitinib, the two main therapies for AML and MPN. Bioinformatics analysis identified genes whose expression was correlated with CD45 expression such as JAK2, ACTR2, THAP3 Serglycin, and PBX-1 genes, as well as licensed drugs (alendronate, allopurinol, and balsalazide), which could be repurposed as CD45 inhibitors which effectively increases sensitivity to cytarabine and ruxolitinib at low doses. Therefore, CD45 inhibition could be explored as a potential therapeutic partner for treatment of myeloid malignancies in combination with chemotherapy such as cytarabine especially for elderly patients and those showing chemotherapy resistance.

**Supplementary Information:**

The online version contains supplementary material available at 10.1007/s00277-023-05520-y.

## Introduction

CD45 is a trans-membrane glycoprotein with a molecular weight of 180–240 kDa expressed on all haematopoietic cells, except erythrocytes and platelets in addition to its expression on all human tissues. On T and B lymphocytes, CD45 forms non-covalent bonds and associates with various cell membrane molecules such as CD1, CD2, CD3, CD4, and galectin-1 [1]. However, the specific function of CD45 is still poorly understood. To elucidate the biological physiology of CD45 in the development of haematopoietic stem cells, membrane immunofluorescence technology was used to investigate its expression on human malignant cell lines [1, 2]. The study showed that CD45 expression is heterogeneous in B-lineage cells with increase on cell differentiation and maturation. In comparison, myeloid and T-cell leukaemia cells showed stable expression. The lack of CD45 expression in a case of T-cell lymphoma could indicate the vital role that CD45 plays in differentiation and activation of haematopoietic cells [1].

Acute myeloid leukaemia (AML) is treated with standard regimens of chemotherapy and stem cell transplantation. Chemotherapy begins with an induction treatment, a combination of drugs, such as cytarabine and daunorubicin [3]. However, it is not tolerated well in older patients and as most AML cases occur in patients over the age of 60. It would be beneficial to introduce targeted therapies which have fewer adverse side effects and are more specific in their action. In addition, relapsed or refractory leukaemia is a challenge to treat and as there are few effective therapies. One way to approach this problem is to develop new therapies based on the strategy of drug-repurposing technology. For instance, thalidomide (anti-emetic drug used for the treatment of nausea) was found to be an effective treatment of multiple myeloma [4]. Pre-purposed drugs characterized by defined toxicity profiles as they are already in clinical use, therefore, are taking less time to reach the market. The drug-repurposing principle has been applied for anti-leukaemic therapies in well-known patient sub-groups. The Blood Cancer Research Group in Belfast has developed a screening strategy of Multiplex Screening for Interacting Compounds in AML (MuSICAL) based on combining approved drugs. The advantage of this approach is to extend the possibility of investigating synergistic combinations of compounds which can be used as a potential therapy. In addition, it reduces replication, redundancy, and cost. This resulted in exploring a triple combination of glimepiride, a sulfonylurea; pancuronium dibromide, a neuromuscular blocking agent; and vinblastine sulphate, a vinca alkaloid, as a potential therapy for paediatric AML. This drug combination strategy of drug repurposing could either provide alternative therapies or enhance efficiency of existing therapies [4–6].

Herein, we identified new potential CD45 inhibitors through bioinformatic analysis and suggest that treating myeloid cells with a combination of CD45 inhibitor drugs (alendronate, allopurinol, and balsalazide) and low-dose cytarabine would have a beneficial therapeutic effect with less toxicity.

## Methods

### Cell culture

All cell lines were obtained from the Deutsche Sammlung von Mikroorganismen und Zellkulturen (DSMZ) (Germany). The cell lines studied were HEL; OCI-AML3; OCI-AML2, THP1, SKM, HL-60, NB4, and UT-7 cells, originating from humans with myeloid leukaemia, and others REH and NALM-6 from precursor B-cell acute lymphoblastic leukaemia patients. Primary AML and MPN cells used were derived from patient samples submitted to the NI Biobank and normal blood harvests were obtained from the NI Blood Transfusion Service. All cell lines were maintained in Roswell Park Memorial Institute (RPMI) 1640 (Thermo Fisher Scientific, UK) supplemented with either 10–20% foetal bovine serum (FBS; Thermo Fisher Scientific, UK) and 100 µg/mL penicillin–streptomycin (Thermo Fisher Scientific, UK). Cell lines were authenticated using short tandem repeat (STR) profiling carried out by the suppliers, incubated at 37 °C, under 5% CO_2_, and sub-cultured every 3–4 days to maintain exponential growth.

### Western blotting

Following cell treatments, the whole cell lysates were collected after mixing cell pellets with 100 μL of radio immune precipitation assay buffer (RIPA). Protein sample concentration was determined by using the bicinchoninic acid protein assay kit (Pierce; USA). Each sample (20 μg) was electrophoresed through a 4–12% sodium dodecyl (SDS)-polyacrylamide gel (Life Technologies, UK), transferred onto a nitrocellulose membrane (Hybond-C, Amersham; UK), and probed with antibodies which include CD45 (PA5-95,187), JAK2 (MA5-15,632), ACTR2 (A305-216A-M), THAP3 (PA5-50,841), PBX-1 (PA5-29,674), and SEG (PA5-51,462), from ThermoFisher Scientific, UK. Other apoptotic antibodies such as caspase 3 (9662S), caspase 9 (9502S), cleaved caspase 9 (7237S), PARP (9542S), and cleaved PARP (5625S) were purchased from Cell Signaling, UK. Rabbit anti-rat HRP (ab6734) and rabbit anti-mouse HRP (58802S) antibodies were purchased from Abcam-UK. CRISPR-Cas9 (NBP2-36,440) was purchased from Novus. GAPDH was used as a loading control (Sigma; UK). Levels of protein expression were assessed using Pierce ECL western blotting detection kit (Thermo Scientific; UK). Membranes were scanned using benchtop G:box (Syngene; UK) and density was calculated using the image J program (http://rsbweb.nih.gov/ij/) incorporating correction of loading controls.

### Flow cytometry

Cell pellets were collected by spinning at 200 × g for 5 min at 4 °C. Cells were re-suspended in 250 µl blocking agent (PBS/0.2% BSA/0.1% sodium azide) and stained with either IgG control (PE Mouse IgG1 Isotype Ctrl -Biolegend-400111) or the antibody stained for its corresponding antigen CD45 (PE anti-human CD45 Antibody Biolegend-304008) at 4 °C in the dark for 60 min. Five milliliter buffer was then added, and samples were spun at 200 × g for 5 min at 4 °C. Buffer was discarded, and the cells were re-suspended in 300 µl PBS and kept at 4 °C in dark until analysis. Mean fluorescent intensity was quantified using the LSR II flow cytometer (BD Biosciences-UK) and data acquisition was performed with FACSDiva software.

### Polymerase chain reaction (PCR)

RNA was isolated and purified from cultured cells using RNeasy Mini Kit (Qiagen, Germany). RNA concentrations were estimated by NanoDrop ND-100 spectrophotometer (Thermo Scientific, USA). Reverse transcription (RT) from total RNA was performed to synthesize cDNA using the high-capacity cDNA reverse transcription kit (Applied Biosystems, USA). RT-PCR was then performed using SYBR green chemistry (Roche, UK) and the 7900HT Fast Real-time PCR platform. CD45 (PTPRC) Human qPCR Primer Pair (NM_002838) was used (Forward Sequence CTTCAGTGGTCCCATTGTGGTG; Reverse Sequence CCACTTTGTTCTCGGCTTCCAG), while QuantumRNA™ Universal 18S Internal Standard (AM1718) was used as endogenous housekeeping control gene (primer sequence for 18SrRNA is: 5′-CCAATTACAGGGCCTCGAAA-3′). Plates were inserted into a thermal cycler: 95 °C for 5 min, 45 cycles of 95 °C for 10 s and 60 °C for 20 s and 72 °C for 1 min. Data analyses were done using the ΔCT method.

### CD45 knockdown by nucleofection

The cell lines were grown and cultured 2 days before nucleofection at 1 × 10^6^ cells/100 µl. Next day, cells were combined with 5 µl of positive control vector pmaxGFP® which encodes the green fluorescent protein (GFP) or 2 µM siRNA and 100 µl the appropriate cell type–specific Nucleofector® solution and transfer to an Amaxa certified cuvette. The cuvettes were inserted into the Nucleofector® II Device (Lonza-UK) and nucleofected using pre-set SMG-THP1 program. The cuvettes were then rinsed with 500 µl pre-warmed medium and then transferred into 6-well culture plate and incubated at 37 °C. The maxGFP® expressing cells were analyzed by EVOS fluorescence microscopy (ThermoFisher Scientific, UK) to monitor transfection efficiency. Transfection has been assessed by PCR and western blotting after 48 h of incubation.

### CRISPR CAS9 CD45 over-expression

By using Horizon kit from Dharmacon-UK, manufactor protol was followed for optimization of best blasticidin antibiotic concentration, CD45 plasmid DNA preparation from glycerol stock, lentiviral transfection, and creation of a stable CAS9 expressing cell line. Lentiviral synthetic guide RNA (sgRNA) glycerol stocks, CAS9 lentiviral particles were transfected by sgRNA into NALM-6 and UT-7 cell lines. This was done by mixing 5 µl of the viral particles into a 100 µl of 2 × 10^5^ cells in an Opti-MEM medium containing the optimized blasticidin concentration and incubated at 37 °C for 4 h, after which a 900 µl of medium with serum but no antibiotic was added and incubated with a cell control for 48 h. This was followed by transfection of CD45 plasmid DNA into CAS9 + cells by mixing 1 µl from each of CD45 plasmid DNAs into 16 µl of complete medium and in another tube 9 µl of DharmaFECT kb, transfection reagent with 11 µl medium, left incubated at RT for 5 min, mixed and left for a further 20 min at room temperature (RT). Subsequently, a 160 µl medium was added to the mixture which was then added to 1 ml CAS9-positive cells and incubated at 37 °C for 48 h along with control cells. CD45 over-expression on NALM-6 and UT-7 cells was validated by western blotting and RT-PCR. The following figure illustrates the elements that construct the vector of the Edit-R Lentiviral sgRNA used in this experiment.

### In vitro* drug treatments*

Cytarabine was purchased from Sigma-Aldrich-UK. Ruxolitinib was purchased from Selleckchem-UK. Recombinant Interleuken-6 was purchased from InvivoGen-UK. All other drugs including sodium orthovanadate—CAS 13721–39-6, alendronate, allopurinol, and balsalazide were purchased from Sigma-Aldrich-UK. Compounds were diluted with Dimethyl Sulfoxide (DMSO) (Sigma-Aldrich, UK) and added to cells to generate the desired drug concentration. Cells treated with 0.1% DMSO were used as a vehicle control. Following treatment, cells were incubated for 72 h in a humidified incubator at 37 °C supplemented with 5% CO_2_. All combination drug screening treatments were performed using the Echo liquid handling technology (Labcyte-UK). Synergism was determined using combinational index (CI) value was calculated using the computer software CompuSyn-UK. CI values < 1 indicate synergy; CI values = 1 indicate an additive effect and CI > 1 indicate an antagonistic effect.

### Cytotoxicity studies using Cell Titer-Glo®

To investigate the effects of gene silencing, over-expression, small molecule treatments, and induction of stimulated signalling pathways on cellular viability and proliferation, the Cell Titer-Glo® Luminescent Cell Viability Assay (Promega, Madison, USA) was employed. Fifty microliters of homogenous lysis reagent was mixed in equal portion with 50 µl of cell culture in triplicate in a white microtitre plate. Each plate was incubated at room temperature for 30 min on the orbital shaker (Stuart Scientific, Staffordshire, UK). Luminescence was measured using a Synergy HTX Multi‐mode Microplate reader (Biotek, Winooski, VT, USA).

### Caspase-Glo™ Assay

Caspase-Glo™ Assay (Promega: UK) quantifies both caspases 3 and 7 activation which is indicative of apoptosis. Following treatments, 50 µl of caspase reagent was mixed in equal portion of cell suspension in 96-well white plate. Each plate was incubated at room temperature for 45 min on the orbital shaker (Stuart Scientific; UK). The luminescence signal was detected using Synergy HTX Multi-mode Micro-plate reader (Biotek, Vermont, USA).

### Bioinformatic analysis of CD45 correlated genes and drugs

Genes were selected following a many-to-one *t*-test in the Partek genomic suite to examine the relationship between CD45 and the genes selected. Those with the highest *R* value showed that they had the same expression directional as CD45. Selected top genes then introduced to Quadratic software as signatures to explore genes and drugs correlated to CD45 expression. Selected genes/drugs were validated using western blot analysis and PCR.

### Statistical analysis

One-way ANOVA or unpaired *t*-tests were performed between experimental groups on all data using GraphPad Prism 8 software (GraphPad, California, USA). Differences of *p* < 0.0001 (****) indicated very strong significance, *p* < 0.001 (***) indicated strong statistical significance, *p* < 0.01 (**) significant, *p* < 0.05 (*) a weak statistical significance, and *p* ≥ 0.05 (ns.) shows no statistical significance.

## Results and discussion

### Heterogenicity of CD45 expression and response to cytarabine

The treatment of MPNs and AML has improved over recent years. Cytarabine was first discovered in the 1960s as a chemotherapy agent targeting hematological cancers [7]. Cytarabine mechanism of action is based on the inhibition of DNA and RNA polymerases and nucleotide reductase enzymes which are vital for DNA synthesis [8]. However, cytarabine has many toxic effects including anemia, fever, pneumonia, leucopenia, conjunctivitis, and disturbance of the gastrointestinal (GI) tract. Moreover, AML patients seem to vary in their response to cytarabine; this might be due to differential expression of various genes in patients. This urges the need to find alternative treatments for AML based on differential gene expression levels. One gene that is poorly studied in the context of drug treatments is CD45, despite its crucial role AML and MPN development and maintenance.

**Fig. 1:**
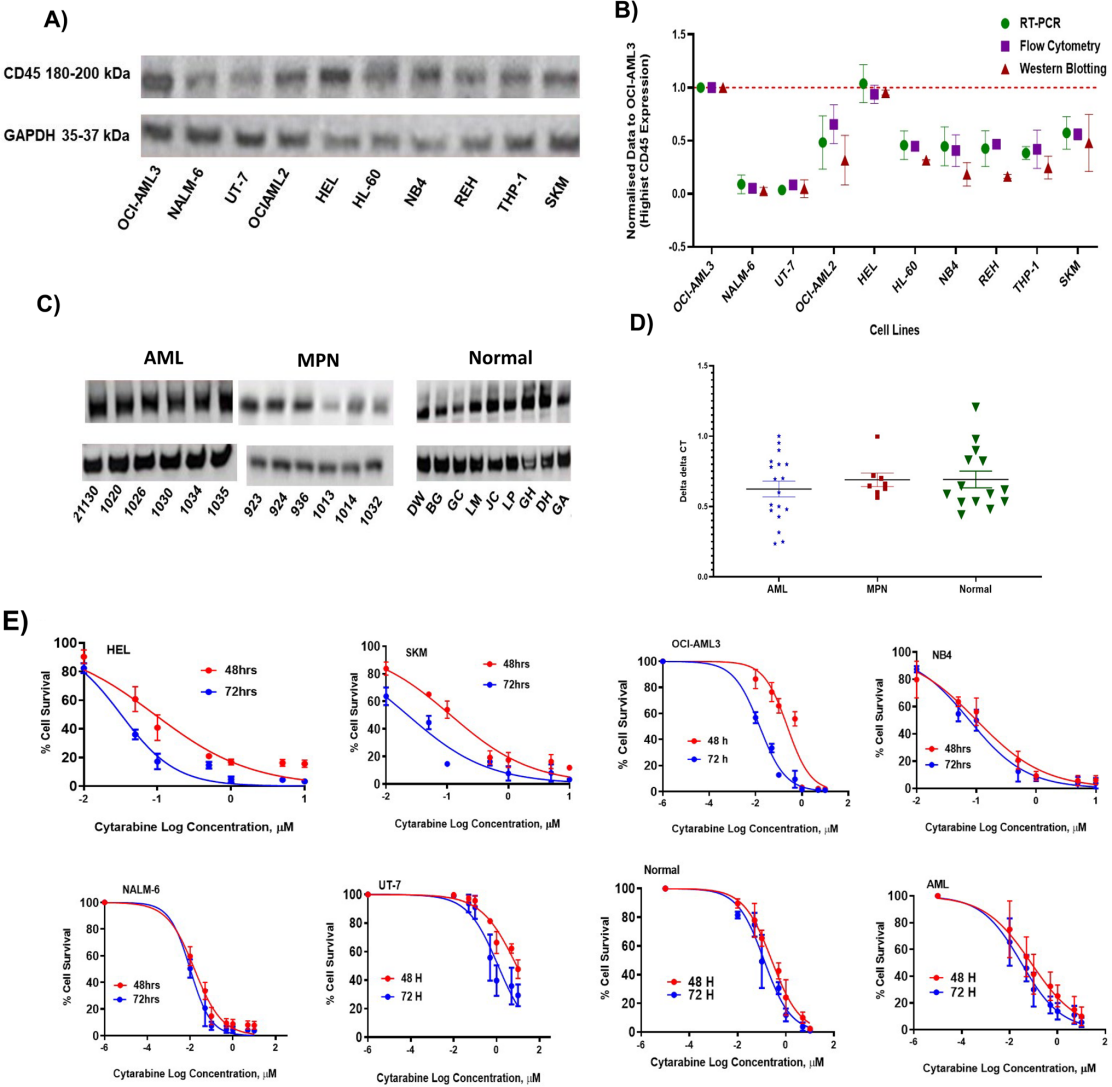
Assessment of CD45 expression on myeloid leukaemia cells and their response to cytarabine. Western blot analysis of CD45 on cell lines (**A**). A scatter blot was performed to facilitate a clear comparison of results obtained by PCR, flow cytometry, and western blotting (**B**) showed OCI-AML3, and HEL cells have a high CD45 expression, while NALM-6 and UT-7 cells characterise low CD45 expression. Western blots of CD45 expression on cells from normal individuals, AML, and MPN patients (**C**), and further validation of ex vivo CD45 expression by RT-PCR (**D**). Cytarabine dose response curves on myeloid leukaemia cells (**E**). The error bar represents standard deviation of the mean from three repeats (*n* = 3, SEM); ex vivo experiments represent peripheral blood cells of normal individuals (14 samples), AML (18 samples), and MPN (8 samples) patients

Our results showed differential expression profiles characteristic of each cell line with OCI-AML3, representing the highest value of CD45 expression, while UT-7 cells showed lowest CD45 expression value (Fig. 1A). This was confirmed using three different techniques (Fig. 1B). CD45 expression of patient peripheral blood cells was also heterogeneous (Fig. 1C and D). It has been previously reported that myeloid leukemia cells exhibited different ratios of CD45 expression, with precursor cells in the bone marrow and leukemia cells expressing low and intermediate values of CD45, while lymphocytes and monocytes commonly expressed high levels of CD45. It has been found that CD45 expression on clonal plasma cells within the bone marrow may be an adverse prognostic marker in patients with multiple myeloma, as it is associated with higher proliferation [9, 10]. In addition, children with precursor T-cell ALL are at risk of relapse due to high CD45 expression [11].

Next, we characterized the in vitro effects of cytarabine (0.01 to 10 µM) in a clinically relevant cell line model of myeloid cell lines and primary cells of myeloid leukaemic patient samples (HEL, SKM, OCI-AML3, UT-7, NALM-6, and NB4), as well as AML patient peripheral blood cells and cells from normal individuals. Dose responses were monitored at 48- and 72-h time points. Cytarabine dose response curves among the tested cell lines (Fig. 1E) showed differential sensitivity with IC_50_ values ranged from 0.004 µM (NALM-6) to 1.2 µM (UT-7) following 72-h treatment. This indicates UT-7 cells being the most resistant to cytarabine at all-time points (Table1-supplementary). All CD45 + cell lines showed positive response to cytarabine. For example, HEL and SKM cell lines were very sensitive to cytarabine treatment (0.1 µM IC_50_) and highly express CD45 compared to UT-7 cell line, a human AML cell line (M7) which has a lower CD45 expression and subsequently more resistant to cytarabine with 1.2 µM IC_50_ following 72-h treatment. UT-7 cells are cytokine-dependent capable of growing in interleukin-3 (IL-3), granulocyte/macrophage colony–stimulating factor (GM-CSF), or EPO which has shown to be an anti-apoptotic factor [12, 13].

### Exploring CD45 inhibitors to study cytarabine response

Hence, CD45 has a vital role in leukaemic cell proliferation and development; therefore, its downregulation via gene silencing and/or inhibition can therefore be explored in the context of cytarabine response to AML. Gene silencing using small interfering RNA (siRNA) is one of the most powerful research tools. However, its application to leukemia cells in suspension is challenging due to low efficiency of delivery of the siRNA [14]. Using pre-designed CD45 siRNA, knockdown of CD45 was performed using either lipofectamine or nucleofection and was assessed by PCR and western analysis. There was no satisfactory knockdown of CD45 to proceed to future investigations (40 nM siRNA for 1.0 × 10^5^/ml OCI-AML3 HEL, and SKM cells showed about 50%, 45%, and 35% less CD45 expression compared to the control respectively) (Supplementary Fig. 1). In addition, nucleofection is a harsh method and not clinically relevant. Therefore, we used alternative ways of reducing the level of CD45 expression through small drug inhibitors.

**Fig. 2:**
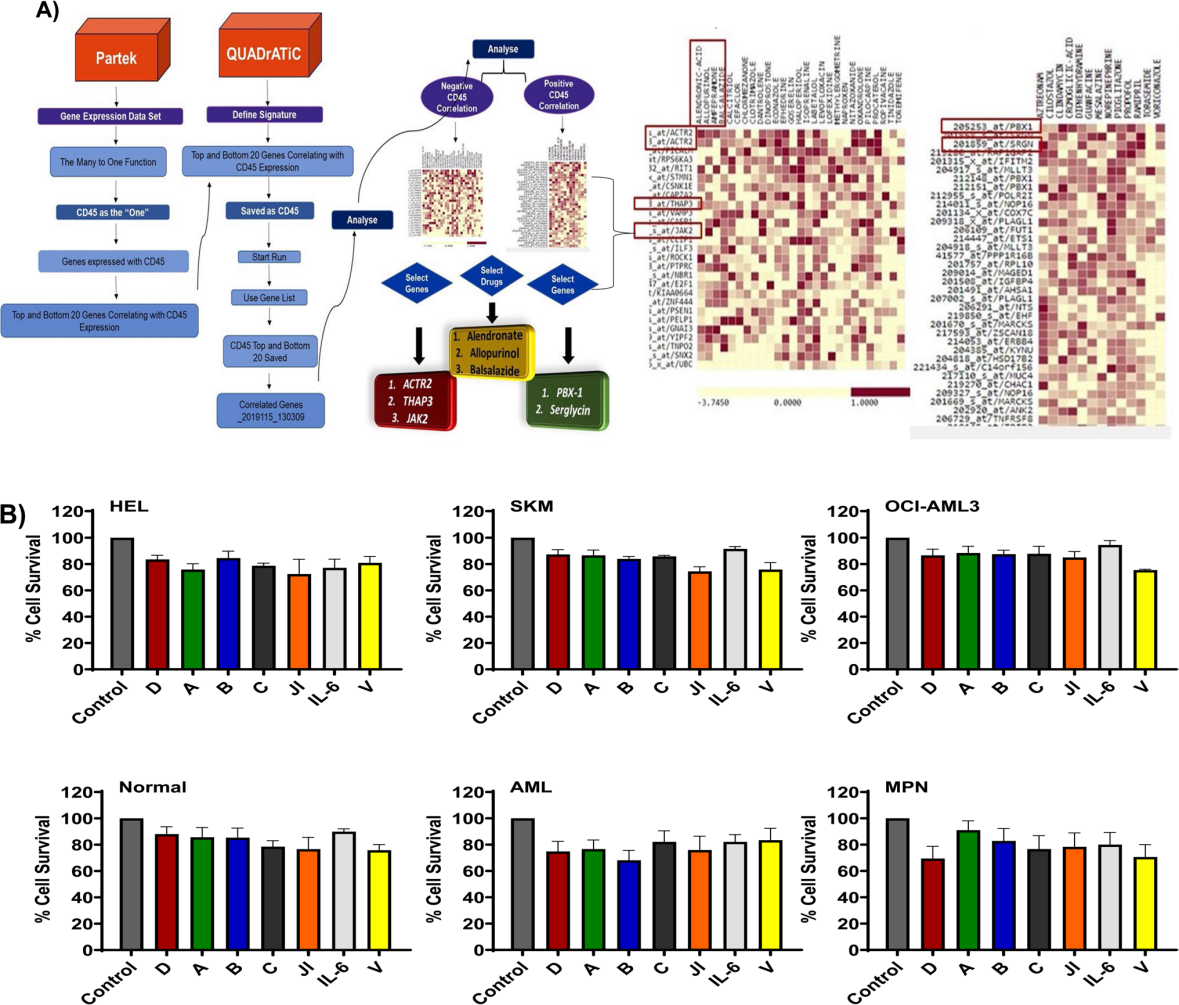
**A** Identification of genes whose expression is correlated with CD45 expression was performed by Partek Genomic Suite software. The genes then further analyzed by Quadratic software which results in visualization of a heatmap showed negative CD45 correlated genes, and another showed positive CD45 correlated genes. This ends with selection of three negative CD45 correlated genes, *JAK2*, *THAP3*, and *ACTR2*, and two positive CD45 correlated genes: *PBX-1* and *Serglycin*. Heatmaps showed identification of a list of genes negatively correlated with CD45 expression, and another of positively correlated genes. **B** Assessment of optimization for non-toxic drug concentrations on normal, AML, and MPN myeloid primary cells and HEL, SKM, and OCI-AML3 cell lines. The drug concentrations used were validated to be non-toxic to the cells as alendronate (D), allopurinol (A), and balsalazide (B) were at 0.01 mM, cytarabine (C) at 0.01 µM, JAK inhibitor (JI) at 1 µM, interleukin 6 (IL-6) at 100 nM, and vanadate (V) at 50 µM. Data is based on three biological replicates and three technical replicates

Queen’s University Belfast accelerated drug and transcriptomic connectivity (QUADrATiC) was developed to investigate gene expression connectivity to data set corresponding to FDA-approved small molecule compounds with the objective of identification of compounds which can be used for repurposing with therapeutic potential [15]. The program is designed to take advantage of multicore computing architectures to provide a scalable solution using the modern concurrent programming paradigm. The QUADrATiC Graphical User Interface (GUI) has been developed to use advanced Javascript frameworks to produce advanced visualization properties for accurate analysis of connections. Following the connectivity mapping analysis, three drugs (alendronate, allopurinol, and balsalazide) that may have a potential role as CD45 inhibitors were identified (Fig. 2). Alendronate is a bisphosphonate applied for the treatment of bone diseases such as osteoporosis [16], while allopurinol is a drug used to reduce levels of blood uric acid and prevent gout and kidney stones [17]. Balsalazide is an anti-inflammatory drug belonging to the aminosalicylates used to treat symptoms associated with ulcerative colitis such as diarrhoea, rectal bleeding, stomach pain, and colonic swelling [18]. JAK2 inhibitor (ruxolitinib) is one of the first-line therapies for MPN patients, and it is a valuable parameter to be included in this study as CD45 is a phosphatase inhibitor of JAK [19]. Therefore, it is important to explore its action on CD45 expression and when CD45 is inhibited. Vanadate was also selected as a potent PTP inhibitor [20] for the purpose of blocking CD45 phosphatase activity rather than inhibiting the whole protein as CD45 plays a vital role in the regulation of the immune system. IL-6 plays an important role in chemotherapy resistance in AML patients due to its activity in stimulating the JAK-STAT pathway, which results in uncontrolled proliferation in leukaemic cells, oncogenesis, and poor prognosis [21]. This indicated that activation of IL-6 could facilitate CD45 expression in myeloma cells. However, little has been reported for myeloid malignancies. Therefore, IL-6 effect on treatment was also investigated in this study. Prior to commencing any experiments, it was vital to optimize doses of CD45 inhibitors, Vanadate, ruxolitinib, and IL-6 that did not cause any significant cytotoxicity by their own (Fig. 2B)**.**

### Effectiveness of repurposed drugs to inhibit cellular CD45 expression

**Fig. 3:**
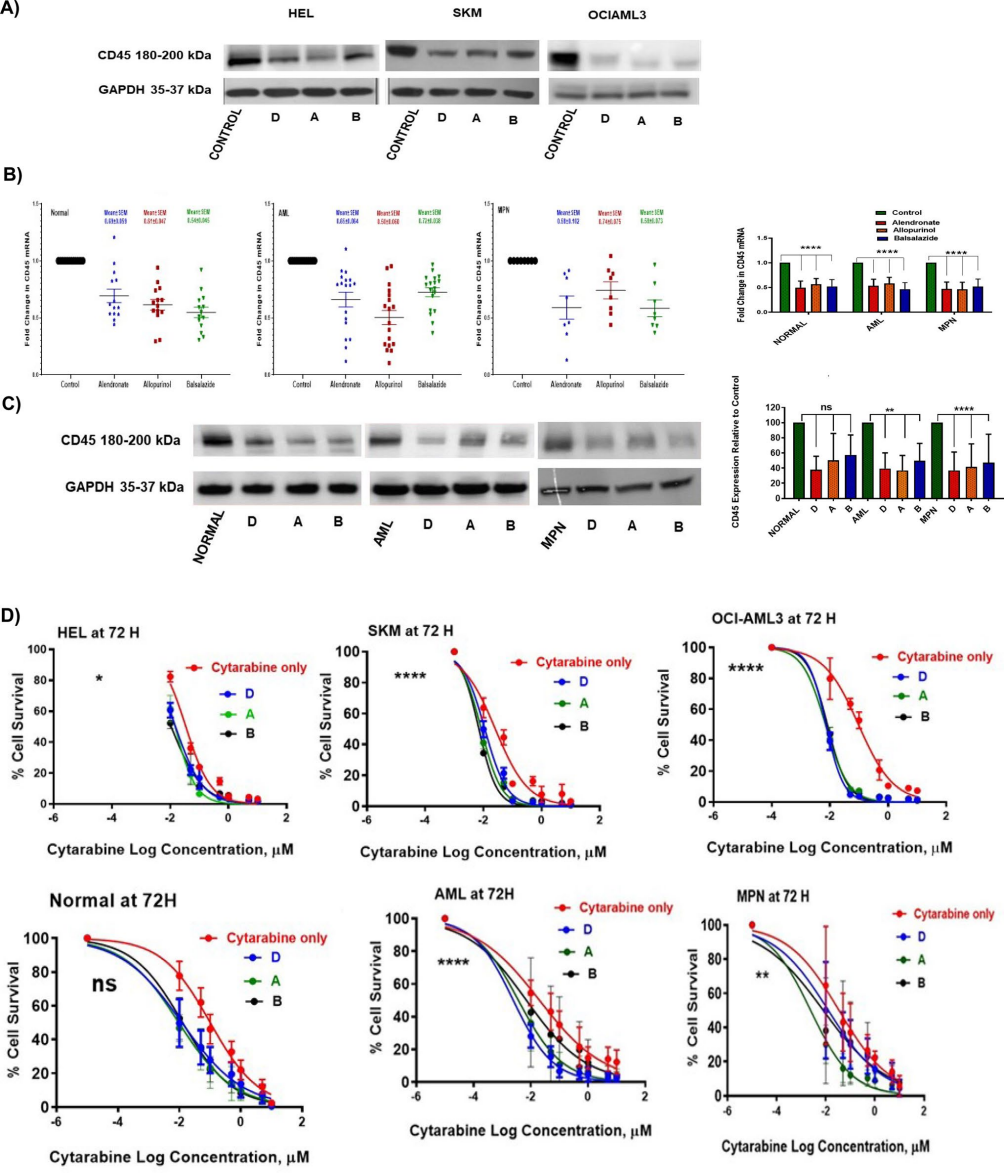
Validation of CD45 inhibitory effect of the selected drugs. CD45 inhibitor drugs at 0.01 mM showed efficacy to inhibit CD45 expression on HEL, SKM, and OCI-AML3 cell lines (**A**), and on primary myeloid leukaemia and normal cells (**B**). Western blot showed significant inhibition of CD45 (*p*-value < 0.0001) on MPN cells and AML cells (*p*-value < 0.004 but non-significant on normal cells confirmed by densitometry of the bands (**C**). Effect of CD45 inhibitor drugs on cellular response to cytarabine on cells treated with CD45 inhibitors (**D**). Abbreviations; D, alendronate; A, allopurinol; B, balsalazide. All experiments represent three independent biological replicates (*n* = 3). The error bar represents standard deviation of the mean from three repeats. Ex vivo experiments represent of peripheral blood cells of normal individuals (14 samples), AML (18 samples), and MPN (8 samples) patients

Three repurposed drugs went through a series of dose optimization to determine the best dosing that was not toxic to the AML cells (Supplementary Fig. 2-A) and primary patient samples (Supplementary Fig. 2-B) and effectively inhibited CD45 (Fig. 3A). Doses were carefully chosen to be clinically relevant by converting used human doses to in vitro doses. Cellular survival was monitored at 48 and 72 h using CTG assay, and protein was extracted from the treated cells and controls at 72 h. Drugs were effective in doses ranging from 0.01 to 0.1 mM with no apparent cytotoxicity. Results showed cellular survival was around 80% by all the treated cells compared to the DMSO control cells. More importantly, the results showed a significant inhibition of CD45 expression in AML primary cells as well as primary cells from normal individuals of about 60–80% (*p*-value < 0.0001), validated by PCR (Fig. 3B). At protein level, western blot analysis showed significant reduction in CD45 level from AML primary cells (*p*-value < 0.004), while primary cells from normal individuals showed a significant reduction (*p*-value < 0.0001) by treatment with 0.1 mM of alendronate (about 70% CD45 expression reduction), but no significant inhibition of CD45 when normal cells treated with the same concentration of allopurinol and balsalazide (Fig. 3C).

### Inhibition of CD45 sensitizes myeloid leukemia cells to cytarabine

Interestingly, when potential CD45 inhibitors were combined with cytarabine, they significantly produced a synergistic effect (Fig. 3D). Cytarabine response curves showed a significant increase in cellular sensitivity at *p*-value < 0.0320 in HEL cells, and *p*-value < 0.0001 in SKM, in OCI-AML3, and in AML primary cells (Fig. 3D), while MPN patient cells showed a *p*-value < 0.008159. Interestingly, Primary cells from normal individuals show no significant effect of CD45 inhibitor drugs (Table 2- supplementary).

### CD45 over-expression had no significant effect on cellular responsiveness to cytarabine

**Fig. 4:**
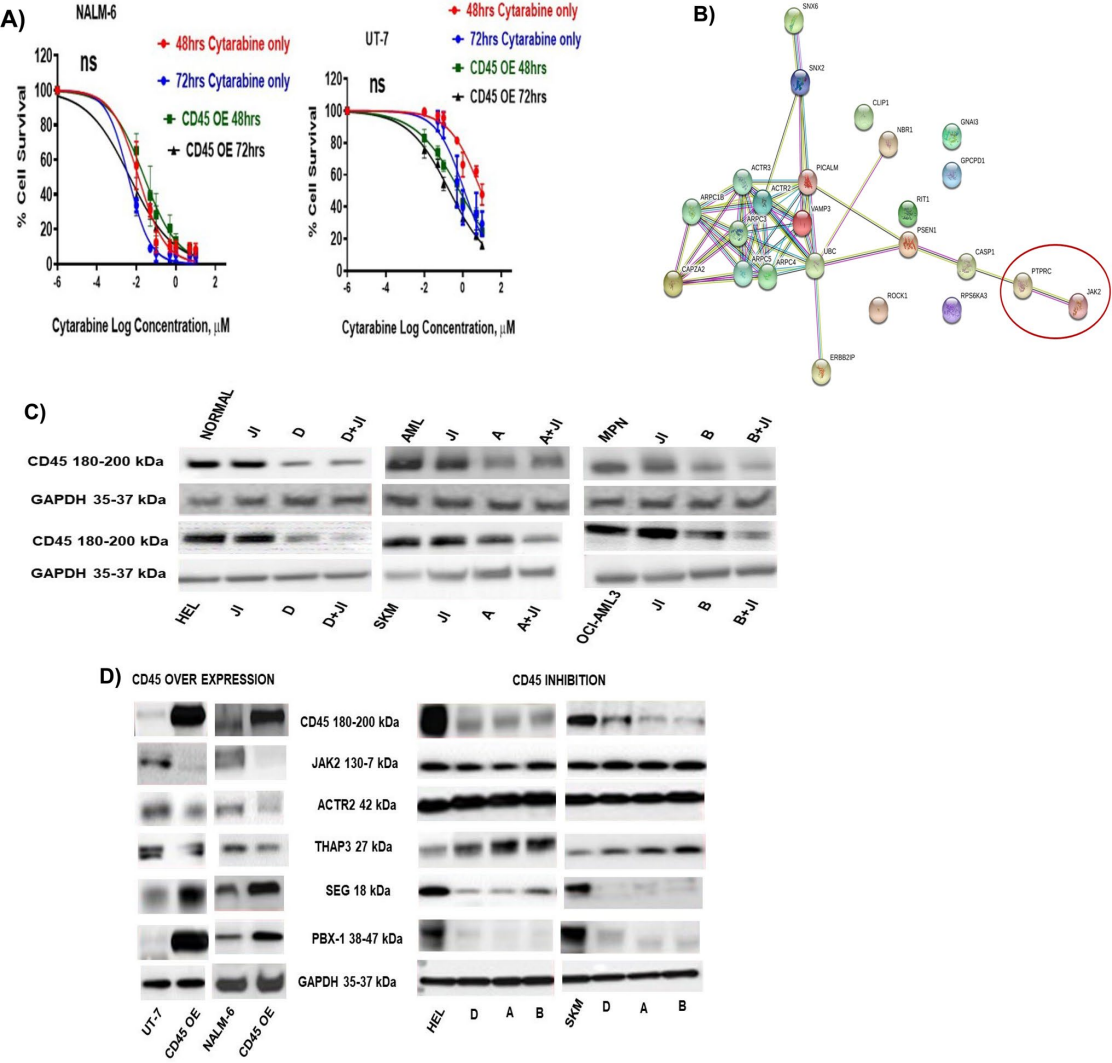
**A** CD45 over-expression has no significant effect on cellular response to cytarabine. CAS9 + UT-7 and CAS9 + NALM-6 cell lines were treated with pre-designed CD45 DNA plasmid for 48 h followed by assessment of CD45 over-expression by PCR. CD45 over-expressed cells were then treated with a dose range of cytarabine. Another set of UT-7 and NALM-6 cells with untreated CD45 expression were introduced with the same range of cytarabine dose for comparison with cell viability being assessed using the Cell Titer-Glo® Assay at 48 and 72 h. **B** Network analysis with STRING software to identify any potential protein interactions. An edge was drawn with up to seven differently coloured lines, representing the existence of the seven types of evidence used for predicting the correlations: a red line marks the presence of fusion evidence, a yellow line represents text mining evidence, a purple line shows experimental evidence, a blue line indicates cooccurrence evidence, a light blue line indicates database evidence, a green line indicates neighbourhood evidence, and a black line represents co-expression evidence. **C** Influence of JAK2 inhibitor on CD45 expression was assessed by western blot on myeloid cells treated with 1.0 µM ruxolitinib (JAK inhibitor) and 0.01 mM of alendronate (D), allopurinol (A), and balsalazide (B) showed that JAK inhibitor alone did not affect CD45 expression, but when combined with any of CD45 inhibitors. **D** Validation of correlation between CD45 expression and the expression of the investigated genes. Western blot showed that expression of *JAK2*, *ACTR2*, and *THAP3* genes was negative to CD45 expression, while *PBX-1* and *SEG* genes were positive to CD45 expression All experiments represent three independent biological replicates (*n* = 3). The error bar represents standard deviation of the mean from three repeats. Ex vivo experiments represent of peripheral blood cells of normal individuals (14 samples), AML (18 samples), and MPN (8 samples) patients

Two days following transfection of UT-7 and NALM-6 CAS9 + cells with CD45 DNA plasmid, and after assessment of CD45 over-expression by PCR, cells were treated with increasing concentrations of cytarabine ranging from 0.01 to 10 µM. Cells were also treated with a DMSO vehicle which served as a control. Dose responses were monitored at 48 and 72 time points. Cell viability was calculated as a percentage of the DMSO vehicle control. Results from the CTG assay showed that CD45 over-expression had no significant effects on cytarabine response curves compared with cells treated with cytarabine only (Fig. 4A). These findings are consistent with the previous studies conducted in myeloid cells studying CD45 phosphatase activity, upregulated by sialic acid, as it inhibits STAT3 transcription factor and facilitates differentiation of macrophages related to tumour progression [22]. In contrast, several studies in solid tumours have shown an association between CD45 expression and reduction of tumour growth [23] but we demonstrated it is not the case for liquid cancers. Taken together, our data suggests that CD45 inhibition could be a potential targeted therapy for myeloid leukaemia patients.

### CD45 correlation with the selected genes

The application of STRING software for the construction of protein interactions among the 40 genes up- and downregulation correlated with *PTPRC* gene encoding CD45 expression which was extracted from Partek genomic suite resulted in the network of interactions represented by Fig. 4B. Obviously, *JAK2* has been reported to interact with CD45 through downregulation which results in inactivation of JAK/STAT pathway which promotes cell apoptosis. This indicates the involvement of CD45 in cell apoptosis [24].

In silico analysis resulted in identification of three genes (*JAK2*, *ACTR2*, and *THAP3*) that were negatievly correlated with CD45 and two genes (*Serglycin* and *PBX-1*) positively correlated with CD45 expression. To validate the correlation between CD45 expression and the expression of the genes identified by quadratic connectivity mapping, CD45 was either over-expressed using CRISPR CAS-9 on UT-7 and NALM-6 cells (Supplementary Fig. 3) or inhibited using repurposed drugs and gene expression was studied at protein level by western blotting. The most important finding from this study was the negative correlation between CD45 expression and *JAK2*. This is in consistent with the results explored by Irie-Sasaki et al. [25]. As *JAK* promotes cellular proliferation through JAK/STAT pathway, inhibition of *JAK* by CD45 might alter cellular proliferation and make cells more susceptible to apoptosis. This confirms the involvement of CD45 in apoptosis. As CD45 expression was validated to be negatively correlated with *JAK2*, then inhibition of *JAK* would not alter CD45 expression but enhance inhibition of CD45 when combined with any of CD45 inhibitor drugs identified. Interestingly, the genes that showed correlation with CD45 expression were also associated with apoptosis such as *THAP3* [26], *Serglycin* [27], and *PBX-1* [28] a trigger to investigate the role of CD45 in apoptosis of myeloid cells. *JAK2* inhibitor maintains CD45 expression at concentrations of 1 µM in all cells studied (Fig. 4C), but it significantly enhanced CD45 inhibitor drugs (*p*-value < 0.0001) to inhibit CD45 expression when combined with 0.01 mM CD45 inhibitor drugs. Therefore, inhibition of CD45 expression causes elevation of JAK/STAT signaling [19, 29], which explains the HEL cells sensitivity to JAK inhibitor compared to SKM cells. UT-7 cells, however, showed the least significant effect of JAK inhibitor due to lower expression of CD45, and, therefore, no significant inhibition of the JAK/STAT pathway. Figure 4D and Supplementary Fig. 4 show that *ACTR2* and *THAP3* were negatively correlated with CD45 expression, while *PBX-1* and *SEG* were positive with CD45 expression. ACTR2 dysregulation is a feature of various types of cancers such as hepatocellular carcinoma and essential thrombocytosis. It is important in cytokinesis, signalling, vesicular trafficking, cellular mechanical process, and to cell shape and motility through lamellipodial actin assembly and projection [30]. This is indirectly indicating the association of CD45 with cellular motility and mobilization. Previously, it has been shown by Shivtiel et al. [31] CD45 over-expression on bone marrow white blood cells was associated with generation of motility in response to stress signals, while CD45 knocked down cells showed decreased granulocyte colony–stimulating factor mobilization and reduction in homing even in response to stromal-derived factor 1, which indicated defective motility. This was due to elevation in cell adhesion induced by decrease in secretion of matrix metalloproteinase-9 and dysregulation of the activity of Src kinase, the main substrate of CD45 [31]. Thus, CD45 regulates movement and retention of progenitor by modulating haematopoietic and non-haematopoietic compartments. The third gene identified which showed negative correlation with CD45 expression was *THAP* domain containing apoptosis-associated protein-3 (*Thap3*). The complex of *THAP3* consists of *THAP1* and THAP3-HCFC1-OGT and it is essential to control the transcriptional activity of *RRM1* gene which encodes ribonucleoside-diphosphate reductase large catalytic subunit M1 [26]. Correlation of CD45 expression with THAP3 is another indication of the role of CD45 in apoptosis. Moreover, CD45 expression is positively correlated with the pre-B-cell leukaemia transcription factor 1 (*PBX-1*) gene which encodes a nuclear protein of *PBX* homeobox family of transcriptional factors which is important in the regulation of osteogenesis, and skeletal modelling and programming. A chromosomal translocation, t(1;19) involving this gene and *TCF3/E2A* gene, is associated with pre-B-cell acute lymphoblastic leukaemia. The resulting fusion protein, in which the DNA binding domain of E2A is replaced by the DNA binding domain of this protein, transforms cells by constitutively activating transcription of genes regulated by the *PBX* protein family [28]. Therefore, this might also suggest the association of CD45 with initiation of transcription of pre-B-cell leukaemia cells as well as the involvement of CD45 in the process of osteogenesis. A study showed that isolated CXCR4 + CD45 − cells enhanced development of an appropriate microenvironment for osteoclastogenesis with a direct effect on the cells expressing SDF-1, CXCL7, and CX3CL1 receptors. Therefore, regulation of CXCR4 + CD45 − cell function might notify therapeutic strategies for diseases involving loss of bone homeostasis [32]. *Serglycin* gene showed a positive correlation with CD45 expression. Researchers suggested that *serglycin* is a crucial factor to promote tumorigenesis in different types of cancer. It is found in the tumour cells phenotype and indicates poor prognosis for disease progression. *Serglycin* functions as an intracellular proteoglycan, but also secreted in the extracellular matrix by tumour cells altering cell properties, oncogenic signalling, and exosomes cargo. *Serglycin* directly interacts with CD44 in addition to its correlation with CD45, and perhaps other cell surface receptors including integrins, inducing cell adhesion and signalling. *Serglycin* also generates a pro-inflammatory and pro-angiogenic tumour microenvironment by monitoring the secretion of proteolytic enzymes, IL-8, TGFβ2, CCL2, VEGF, and HGF. Therefore, *serglycin* stimulates multiple signalling cascades that initiate angiogenesis, tumour cell growth, epithelial to mesenchymal transition, cancer cell stemness, and metastasis. The interference with the tumorigenic functions of *serglycin* emerges as an attractive prospect to target malignancies [27]. Taken together, association between CD45 expression and *serglycin* suggests the importance involvement of CD45 to support the *serglycin* tumorigenesis activity. This should be further explored and validated as a therapeutic target.

### *Enhancing the therapeutic efficacy of cytarabine *via* combination treatment with more than one CD45 inhibitor*

In another approach for the purpose of improving AML patient prognosis, researchers are currently studying drug combinations which have the potential to minimize toxic side effects. Interactions of drugs could be synergistic, antagonistic, or additive based on pharmacological effect for pair or triple drug combination [33].

**Fig. 5:**
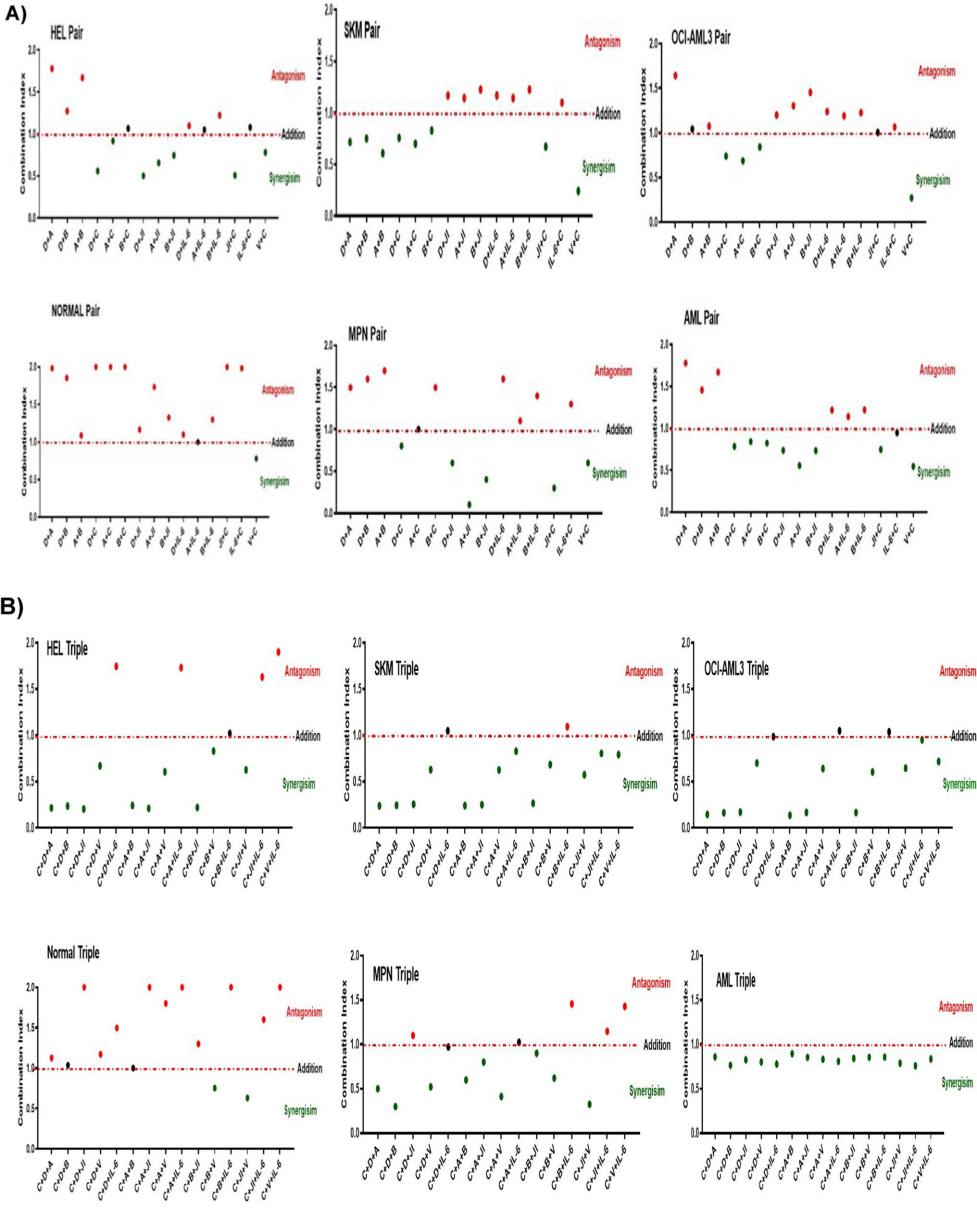
Drug combination experiments on myeloid leukaemia cells. Pair combinations of one CD45 inhibitor drug with IC_20_ cytarabine resulted in a great synergism in HEL, SKM, and OCI-AML3 cells as well as primary cells from MPN and AML patients but were non-toxic to normal cells. Likewise, double drug combination of JAK inhibitor with CD45 inhibitor drugs was non-toxic to normal cells, but can kill HEL cells, MPN, and AML primary cells (**A**). Triple drug combination of two CD45 inhibitors with cytarabine worked synergistically on HEL, SKM, and OCI-AML3 cells as well as primary cells, but non-toxic to normal cells. Additionally, JAK inhibitor was strongly synergized with allopurinol and cytarabine (A + JI + C) in all the cells studied except in normal cells. However, combinations of CD45 inhibitors with IL-6 and/or vanadate with cytarabine worked antagonistically (**B**). Abbreviations: D, alendronate; A, allopurinol; B, balsalazide; C, cytarabine; JI, JAK inhibitor; IL-6, interleukin 6; V, vanadate. All experiments represent three independent biological replicates (*n* = 3). The error bar represents standard deviation of the mean from three repeats. Ex vivo experiments represent of peripheral blood cells of normal individuals (14 samples), AML (18 samples), and MPN (8 samples) patients

Synergy is an important requirement to treat drug-resistant AML patient which is composed of polymerase II inhibitor 1-β-D-arabinofuranoside cytarabine, a deoxycytidine analogue as a single agent or in combination with other chemotherapies [33–35]. Pair combination of cytarabine with CD45 inhibitor was non-toxic to normal cells, but toxic to the other treated cells which indicates that CD45 inhibitor drugs (particularly 0.01 mM alendronate) work synergistically with cytarabine, for instance combination index is less than 0.5 as shown by HEL cell line (originated from MPN patient) (Fig. 5A). This could represent the best novel drug combination therapy for the treatment MPN, and AML patients. Moreover, pairing JAK inhibitor (1 µM) with IC_20_ cytarabine (0.01 µM) worked synergistically on all cells but was non-toxic to normal cells. Also, JAK inhibitor combined with CD45 inhibitor drugs can kill HEL cell line and MPN primary cells, but was non-toxic to SKM, OCI-AML3, and primary cells from normal individuals. This would suggest that inhibition of CD45 by the drugs leads to reduced cellular survival in cells actively dividing by mitosis as in leukaemic cells, while sparing normal less actively dividing cells [36]. However, the addition of 0.01 µM cytarabine with two CD45 inhibitors at 0.01 mM enhanced toxicity and works synergistically to produce a substantial increase in cell death in HEL, SKM, and OCI-AML3 cells. This can be explained by the fact introduced by Yin that any rapidly dividing cells, cancerous or normal, which need DNA replication for mitosis are the most affected by cytarabine [36]. In addition, studies with cytarabine [35–39] have shown that it is active only against dividing cells, and does not cause the death of nondividing cells in vitro even at extremely high concentrations, and can be beneficial in treating myeloid cell leukaemia. The mechanism of action for this drug combination could be causing DNA damage, reduced cell viability, and apoptosis. Therefore, combination of cytrabine with other drugs might be synergistic. Results from this study showed interesting combinations therapeutic potential for future use. It would suggest that treating leukaemia myeloid cells with a combination of CD45 inhibitor drugs and low-dose cytarabine would have a similar beneficial effect.

Thus, combining drugs at low concentrations such as IC_20_ or IC_30_ can be of great benefit to patients avoiding the high toxic concentrations of chemotherapy with its critical side effects. In addition, it would be of great value in overcoming chemo resistence showed by leukaemia cells. Therefore, understanding molecular pathways in each kind of leukaemia cells is vital in order to decide which drug combination is the best and the optimum dosage to be applied for the purpose of achieving the maximum synergistic results and to avoid unnecessary antagonistic effects.

Although IL-6 showed antagonism effect when combined with cytarabine in pair drug combinations (Fig. 5A), adding either, CD45 inhibitor, vanadate or JAK inhibitor as a triple combination caused death to AML primary cells, SKM and OCI-AML3 cells, but not HEL or MPN primary cells (Fig. 5B). This could be explained by the toxic effect of cytarabine which is further promoted by the CD45 inhibitors. Similarly, the triple drug combination of allopurinol with either JAK inhibitor or vanadate and IC_20_ cytarabine was not harmful to normal cells, but toxic to all cells in the study. Those interesting drug combinations could produce an alternative therapy characterized by less toxicity and more efficacy for treatment of myeloid leukaemia patients. Several non-chemotherapy drugs have been found to be non-toxic by themselves; however, they can induce cell death when combined with anti-leukemic therapy such as cytarabine. For example, combining metformin (anti-diabetic drug) and cytarabine was found to be effective in the treatment of relapsed AML [6]. It showed promising results in stimulating the adenosine monophosphate–activated protein kinase pathway dependent on the tumour suppressor LKB1, which indicates tumour-suppressive effects of metformin [6]. Additionally, the combination of pravastatin (anti-cholesterol drug) with idarubicin and cytarabine can be useful for the treatment of relapsed AML [39]. This is based on the fact that leukaemic cells are dependent on cholesterol synthesis for survival, and inhibiting synthesis of cholesterol can lead to increase sensitivity of the cancer cells to chemotherapy [40]. Another repurposed drug was valproic acid, an anticonvulsant in epilepsy and mood stabilizing drug for bipolar disorder. It has shown a synergistic effect when mixed with cytarabine on AML cells, which suggests the anti-leukaemic efficacy of VPA in the treatment of AML by triggering cell apoptosis [41]. Similarly, research conducted by Pigneux et al. [42] used triptolide in combination with cytarabine and showed induction of DNA damage and apoptosis in AML. Thus, development of drug-repurposing technology results in widening of the spectrum of old drugs to be used for the treatment of new diseases, with subsequent alterations in the chemical compositions for the purpose of improving efficiency and reducing necessary dosing and toxicity [42]. This involves further establishment of safety data with global co-operation for cancer therapy development.

### CD45 inhibition on myeloid cell lines enhances apoptosis

**Fig. 6:**
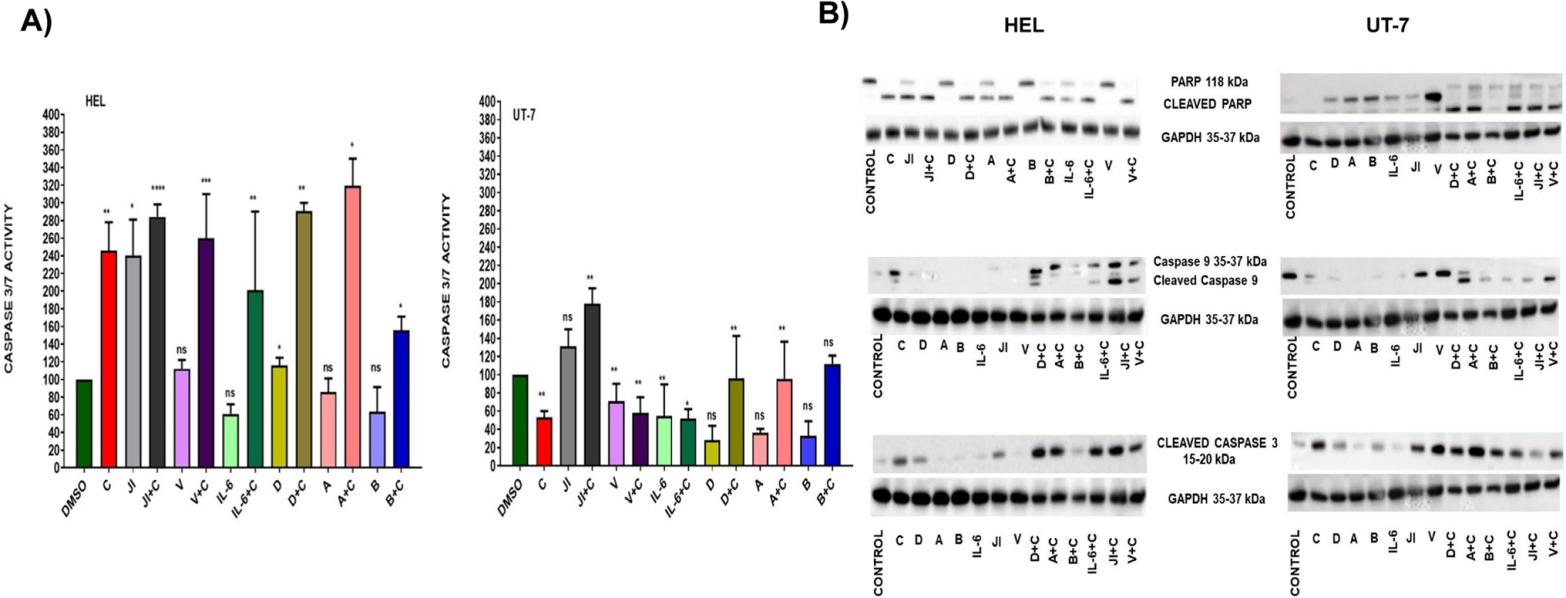
Activation of apoptotic markers in response to combination of cytarabine with CD45 inhibitors. **A** Caspases 3 and 7 activities were significantly (*p*-value < 0.0001) increased by CD45 inhibitors (alendronate, D, allopurinol, A, and balsalazide, B), vanadate (V), JAK inhibitor (JI), and IL-6 combined with IC_50_ cytarabine (C) expression on HEL cells, but not on UT-7 cells compared to DMSO control. **B** Expression of apoptotic markers including PARP and cleaved PARP, caspase 9 and 3, and cleaved 9 and 3. All experiments represent three independent biological replicates (*n* = 3). The error bar represents standard deviation of the mean from three repeats

Two myeloid cell lines were studied; HEL cells as an example of CD45 highly expressed, and UT-7 cells to represent lower CD45 expression to study the effect of CD45 levels to regulate cellular apoptosis. Cells were either untreated or treated with CD45 inhibitors, IL-6, vanadate or cytarabine, or cytarabine combined with CD45 inhibitors. Results showed that caspase 3/7 activity was enhanced by addition of cytarabine (0.01 µM) to CD45 inhibitors, JAK inhibitor, and IL-6 in HEL, but not in UT-7 Cells (Fig. 6A). This could be attributed to the fact that inhibition of CD45 promotes the apoptotic action of cytarabine in HEL cells. Although UT-7 cells have lower CD45 expression, they exhibited resistance to cytarabine as they are growth-dependent to erythropoietin, an anti-apoptotic factor.

PARP expression as a hallmark of apoptosis was stimulated in cells treated by CD45 inhibitors, IL-6, and JAK inhibitor combined with cytarabine. As it is illustrated in Fig. 6B and supplementary Fig. 5, PARP is highly expressed on HEL cells and UT-7 cells treated with CD45 inhibitors as a single agent and cleaved PARP is highly expressed on cells treated by drugs combined with cytarabine shown by both HEL cells and UT-7 cells. The untreated control cells as well as cells treated with cytarabine only showed higher expression of PARP by HEL cells compared to UT-7 cells. This might indicate the association between CD45 expression level on the cells, response to cytarabine which promotes apoptosis indicated by the expression of cleaved PARP as an apoptotic marker. Additionally, treated HEL cells with CD45 inhibitor and cytarabine elevated caspase 9 expression levels, but not on untreated control cells. In contrast, UT-7 untreated control cells were higher in caspase 9 expression compared to HEL cells (Fig. 6B, in supplementary Fig. 5). Obviously, this was a further confirmation of CD45 involvement in apoptosis as HEL cells are high CD45 + and UT-7 cells are low CD45 + . However, low expression of caspase 9 on UT-7 cells treated with cytarabine as a single agent indicates UT-7 resistance to cytarabine. Interestingly, caspase 9 was highly expressed by HEL cells compared to UT-7 cells in response to a combination of JAK inhibitor and cytarabine. This is because HEL cells are *JAK* mutated and are very sensitive to JAK inhibition, while UT-7 cells are not [13]. Furthermore, cleaved caspase 3 is highly expressed on cells treated with CD45 inhibitors, JAK inhibitor, and IL-6 when combined with cytarabine in both HEL cells and UT-7 cells (Fig. 6B, in supplementary Fig. 5). Studying CD45 involvement in apoptosis showed that the high CD45 + HEL cells were more sensitive to apoptosis in response to treatment with CD45 inhibitors and cytarabine than the low CD45 + UT-7 cells. This result is consistent with the one conducted by Nguyen et al. which showed that galectin-1-induced T-cell apoptosis is regulated by CD45, and that cells need CD45 cytoplasmic and extracellular region to induce galectin-1 death [43].

Also, treatment with the phosphotyrosine-phosphatase inhibitor potassium bisperoxo(1,10-Fsupplphenanthroline) oxovanadate(V) enhanced galectin-1 susceptibility of CD45 + T-cell lines but had no effect on the death of CD45 − T-cells, indicating that CD45 inhibitory effect involved the phosphatase domain [43]. This could explain the effect of vanadate-generating expression of PARP on HEL and UT-7 myeloid cells (Fig. 6B).

UT-7 cells are erythropoietin-dependent megakaryoblastic leukemia (M7) and were validated to show antagonistic interaction with cytarabine by Elmoneim et al. [44]. They proved that simultanious treatment of UT-7 cells with cytarabine and 5-aza-2′-deoxycytidine (DAC) resulted in an antagonistic effect, while 72-h pre-treatment of UT-7 cells with DAC increased the sensitivity of UT-7 cells to cytarabine [44]. Hubeek et al. investigated human equilibrative nucleoside transporter 1 (hENT1), the transporter that regulates cytarabine uptake into the cell [45]. For its initial activation, cytarabine depends on deoxycytidine kinase (DCK). Therefore, reduction in cytarabine cellular uptake results from low expression of hENT1 mRNA and *DCK* mRNA with reduced *DCK* activity [46]. This leads to decreased cellular sensitivity to cytarabine in leukaemic cells. It has been found that mutations in *DCK* are responsible for cellular cytarabine resistance [46, 47] and introduction of *DCK* cDNA into *DCK* − / − cells increased cellular sensitivity to Ara-C [48]. In an in vivo study, Veuger et al. investigated alternatively spliced *DCK* mRNAs in leukaemic cells from AML patients with cytarabine resistance, while not found in AML patients sensitive to cytarabine [49]. This indicates that the presence of inactive *DCK* mRNAs could interfere with sensitivity to cytarabine.

## Conclusions

Taken together, this data suggests that CD45 could be a potential alternative therapy that targets myeloid leukemic cells. This approach could help to overcome chemotherapy resistance shown by some myeloid leukaemia patients. Therefore, this molecule may be a potentially useful drug target for the treatment of myeloid leukaemia; however, further studies may be conducted to help fully understand all CD45’s functions before a safe drug could be developed. Given that CD45 is expressed on the majority of haemopoietic cells, including immune cells, it would be beneficial to continue the work with healthy donors when exploring the efficacy of drug combination studies. This will be achieved with the application of more efficient techniques for quantifying drug synergism and toxicity. In addition, increasing sample size of the study will enable a confident prediction of patient’s survival correlated with CD45 expression after adjusting for age, gender, and clinical stages, especially for elderly patients of more than 60 years of age. Moreover, the main goal of this work is to explore a translational potential by application of in vivo experiments and addressing the effect of combining CD45 inhibitors with chemotherapy on other haemopoietic subsets with a great consideration on immune cells.

### Supplementary Information

Below is the link to the electronic supplementary material.

Supplementary file1 (PPTX 4386 KB)
