# Supplementary material for: CD45 inhibition in myeloid leukaemia cells sensitizes cellular responsiveness to chemotherapy
Source: Ann Hematol. 2023 Nov 2;103(1):73–88. doi: 10.1007/s00277-023-05520-y (PMC10761371; doi:10.1007/s00277-023-05520-y)
Supplement: Supplementary file 1 — Supplementary file1 (PPTX 4386 KB) [file 277_2023_5520_MOESM1_ESM.pptx]

## Slide 1
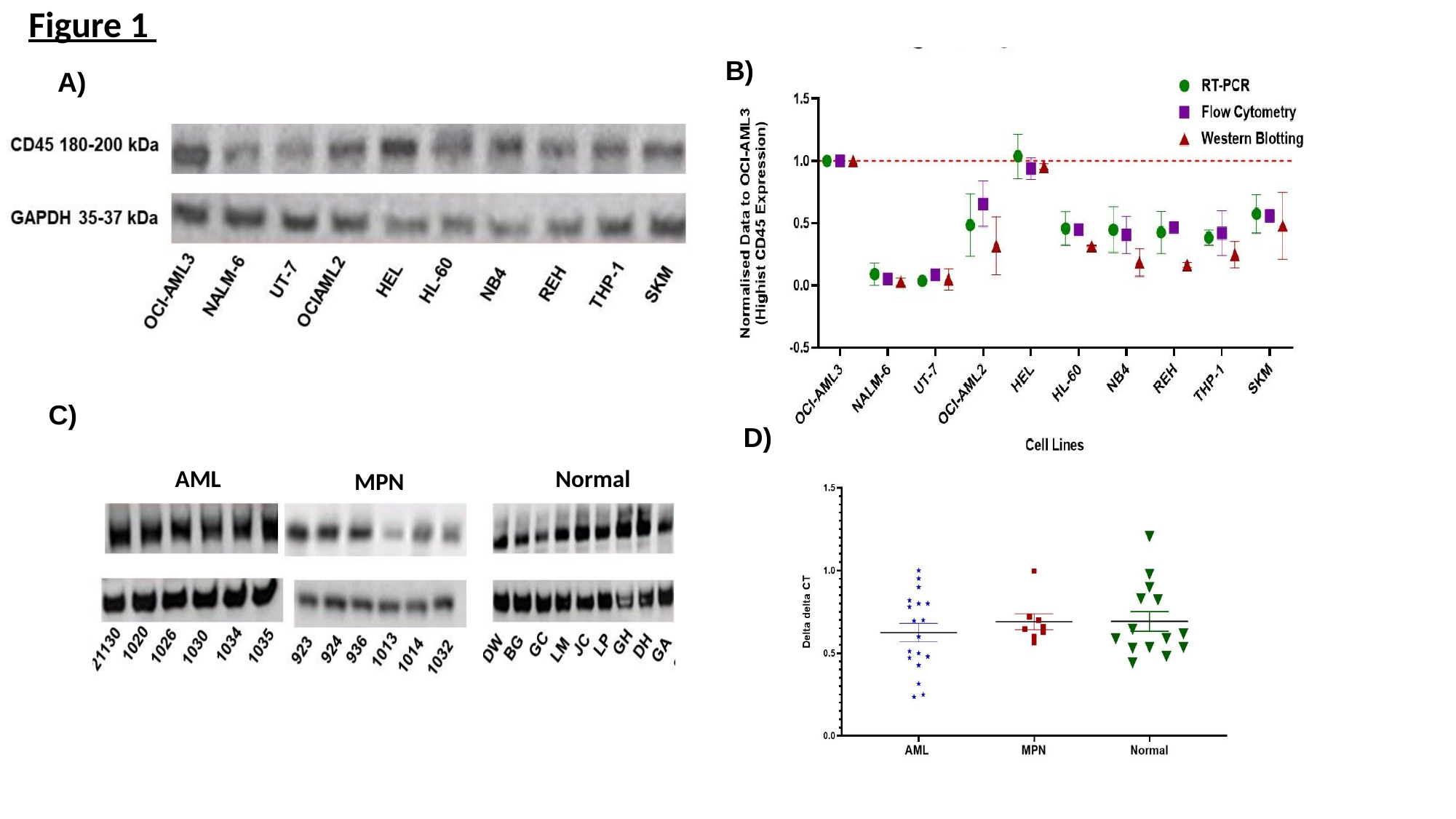

Figure 1
B)
A)
C)
D)
AML
Normal
MPN

## Slide 2
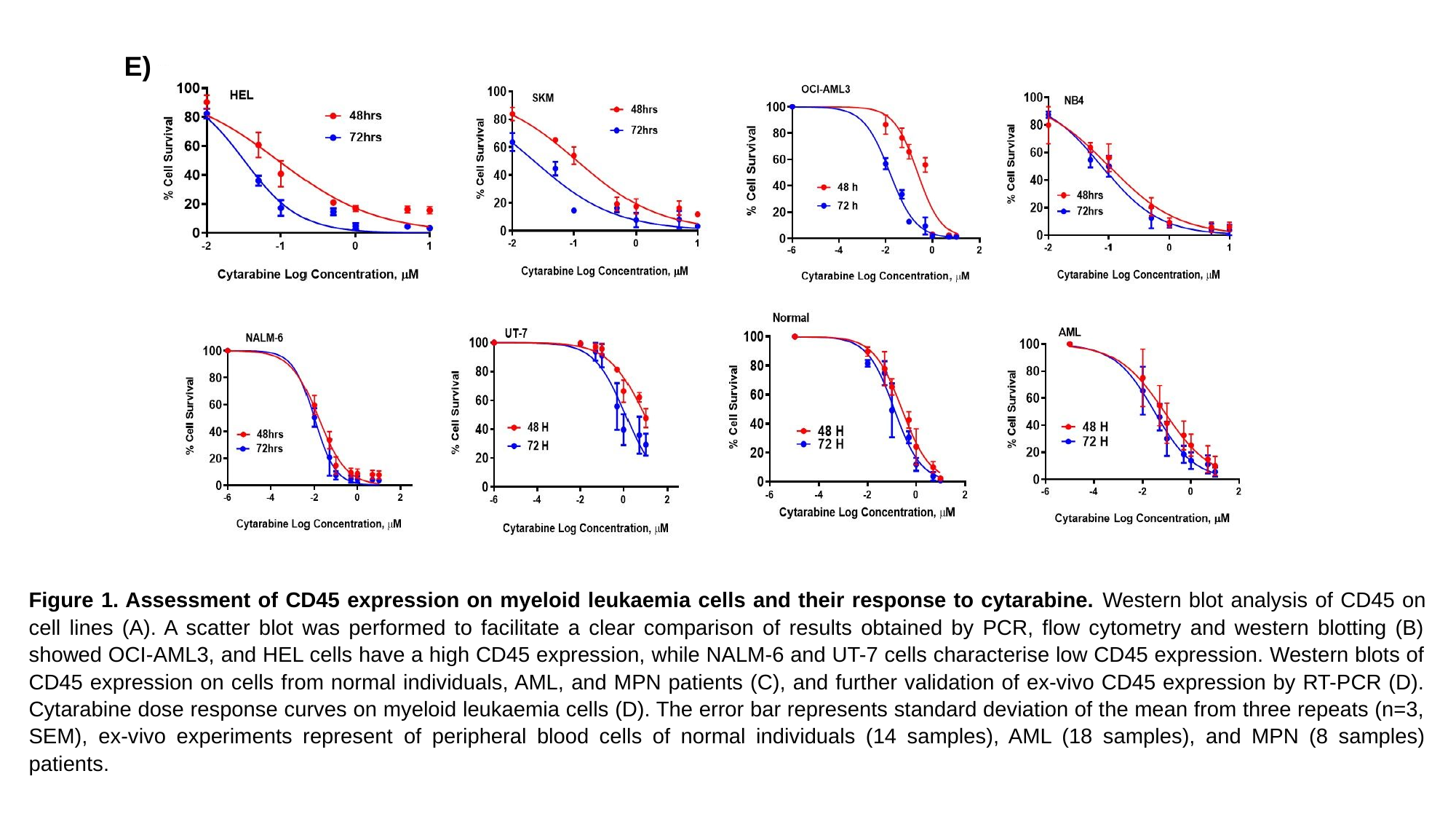

E)
Figure 1. Assessment of CD45 expression on myeloid leukaemia cells and their response to cytarabine. Western blot analysis of CD45 on cell lines (A). A scatter blot was performed to facilitate a clear comparison of results obtained by PCR, flow cytometry and western blotting (B) showed OCI-AML3, and HEL cells have a high CD45 expression, while NALM-6 and UT-7 cells characterise low CD45 expression. Western blots of CD45 expression on cells from normal individuals, AML, and MPN patients (C), and further validation of ex-vivo CD45 expression by RT-PCR (D). Cytarabine dose response curves on myeloid leukaemia cells (D). The error bar represents standard deviation of the mean from three repeats (n=3, SEM), ex-vivo experiments represent of peripheral blood cells of normal individuals (14 samples), AML (18 samples), and MPN (8 samples) patients.

## Slide 3
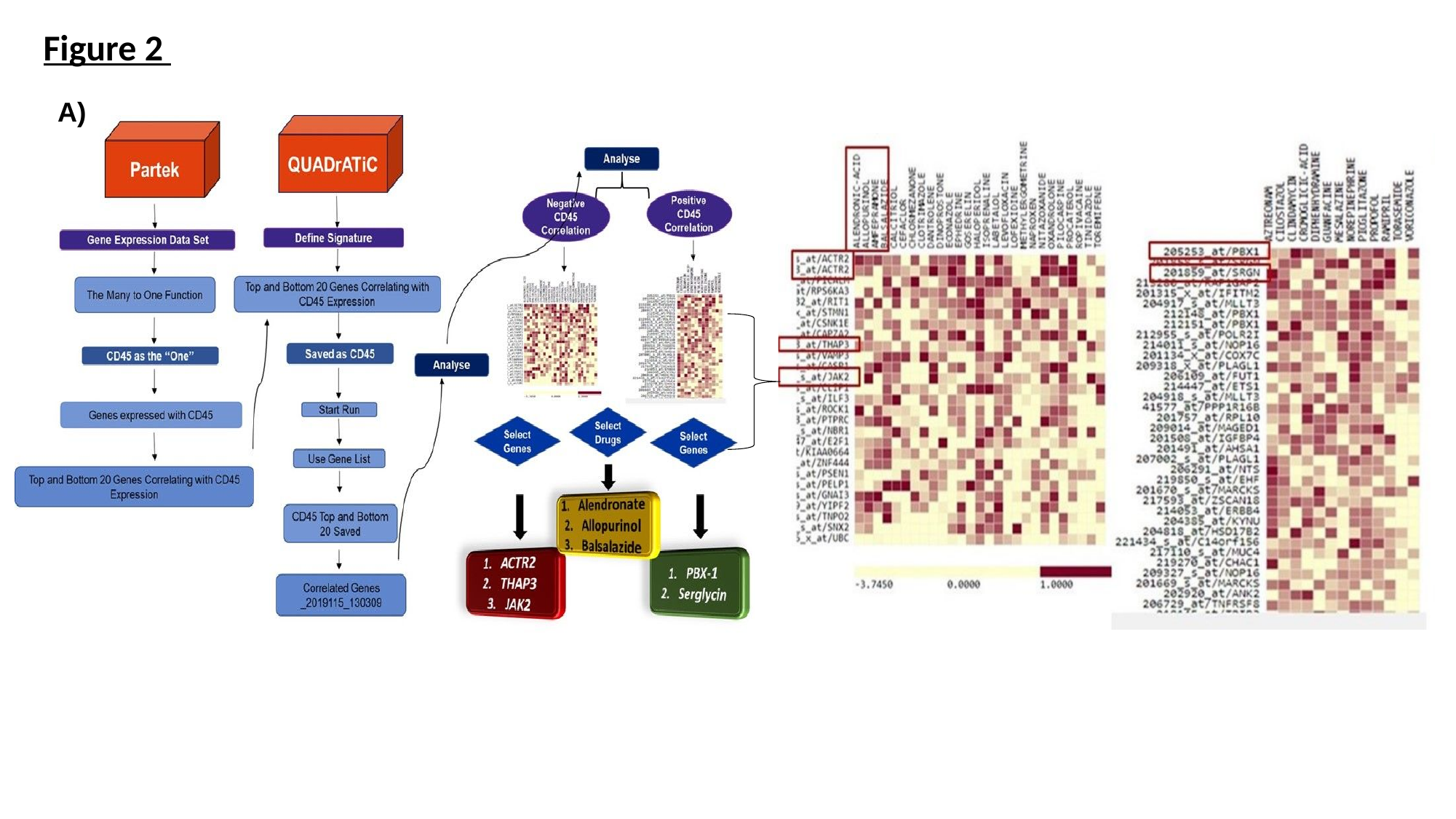

Figure 2
A)

## Slide 4
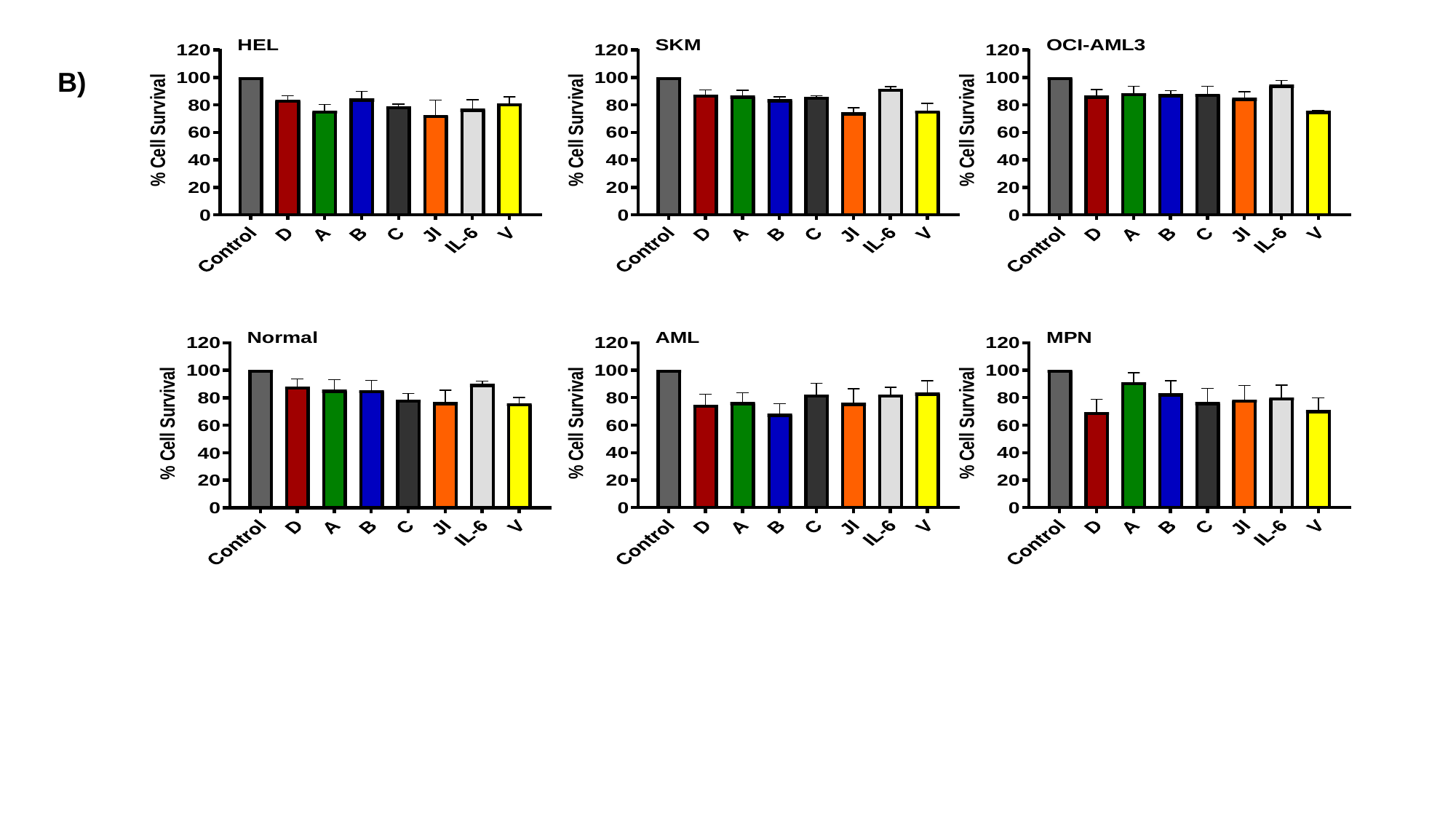

B)

## Slide 5
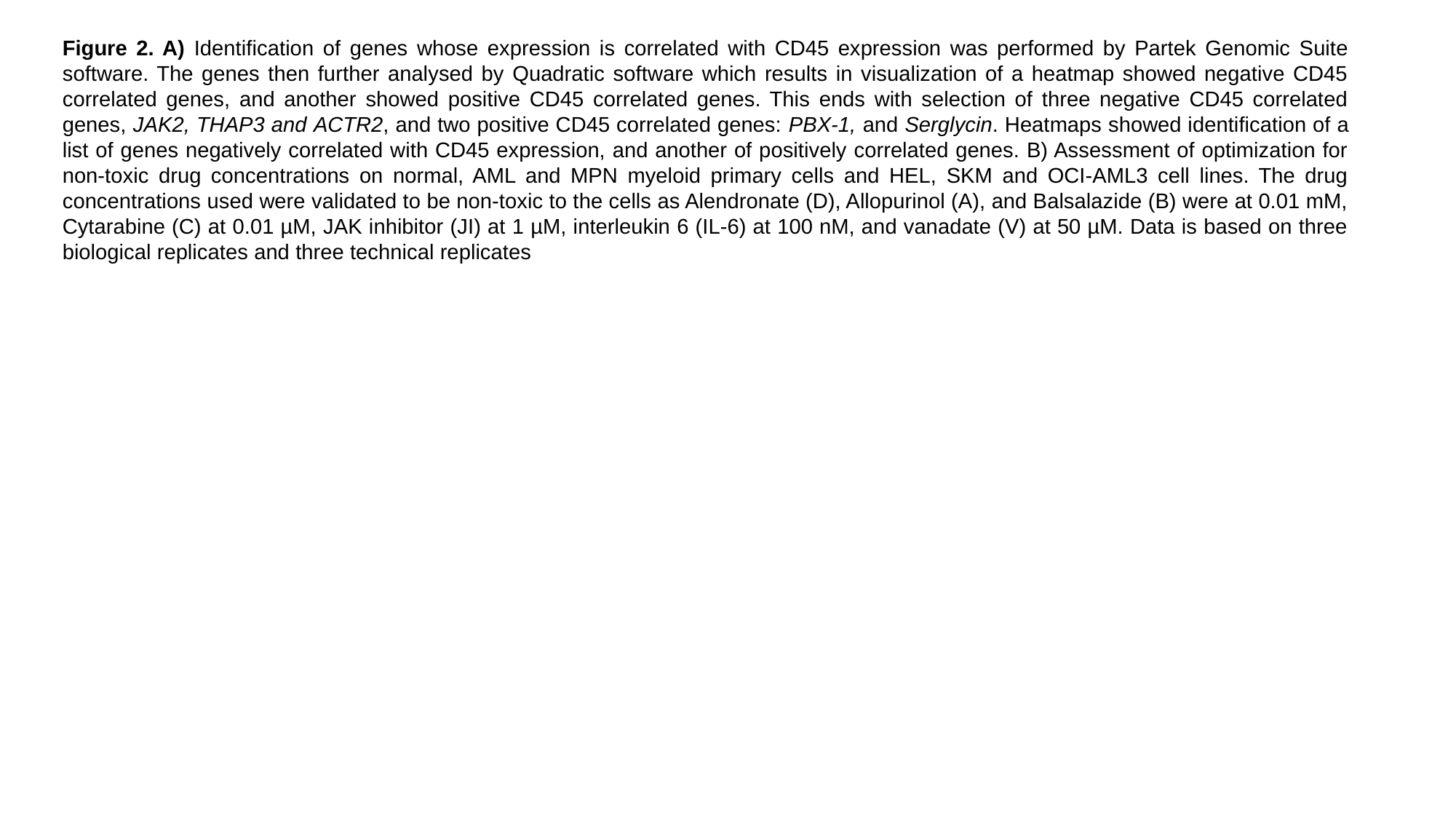

Figure 2. A) Identification of genes whose expression is correlated with CD45 expression was performed by Partek Genomic Suite software. The genes then further analysed by Quadratic software which results in visualization of a heatmap showed negative CD45 correlated genes, and another showed positive CD45 correlated genes. This ends with selection of three negative CD45 correlated genes, JAK2, THAP3 and ACTR2, and two positive CD45 correlated genes: PBX-1, and Serglycin. Heatmaps showed identification of a list of genes negatively correlated with CD45 expression, and another of positively correlated genes. B) Assessment of optimization for non-toxic drug concentrations on normal, AML and MPN myeloid primary cells and HEL, SKM and OCI-AML3 cell lines. The drug concentrations used were validated to be non-toxic to the cells as Alendronate (D), Allopurinol (A), and Balsalazide (B) were at 0.01 mM, Cytarabine (C) at 0.01 µM, JAK inhibitor (JI) at 1 µM, interleukin 6 (IL-6) at 100 nM, and vanadate (V) at 50 µM. Data is based on three biological replicates and three technical replicates

## Slide 6
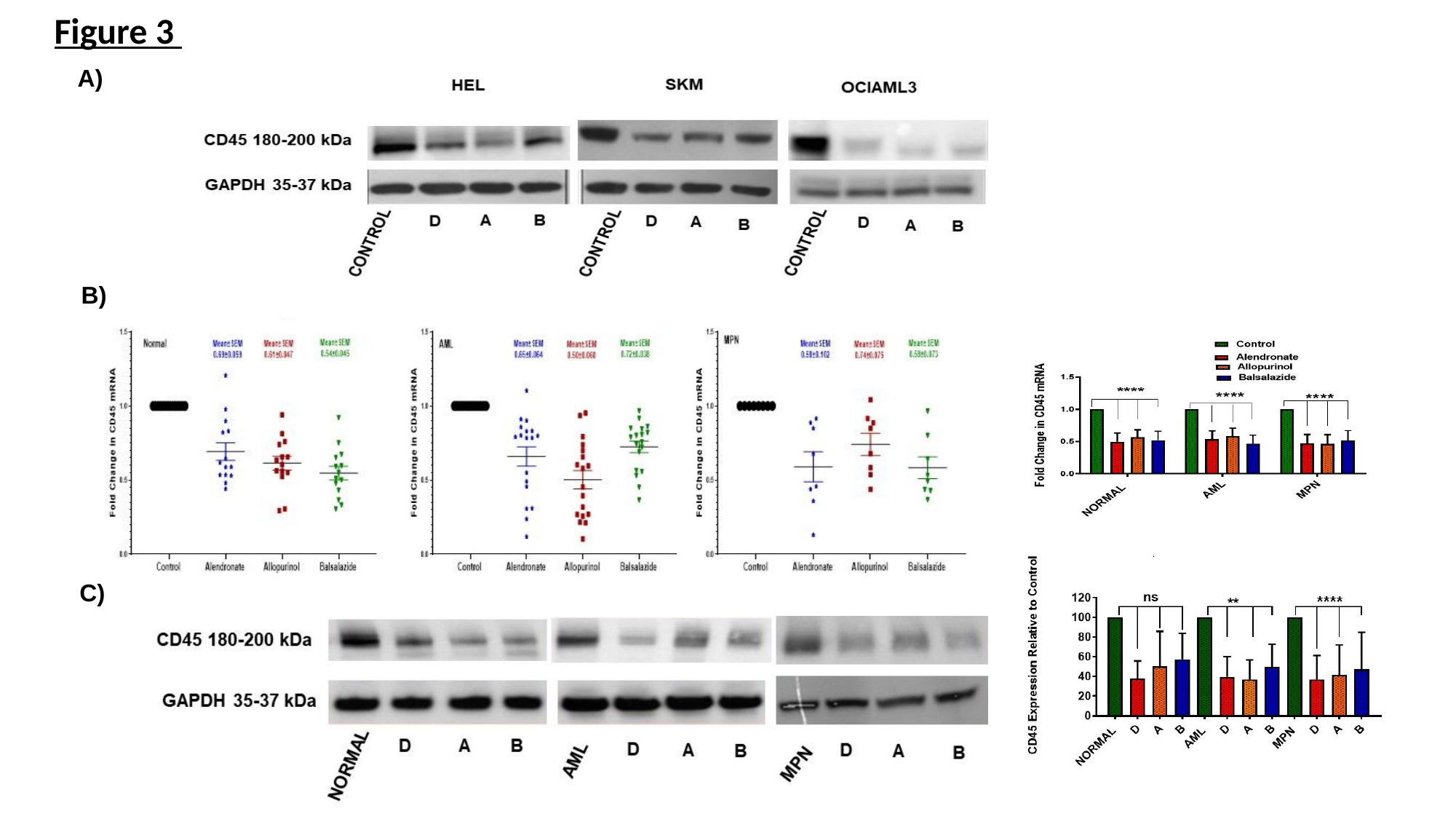

Figure 3
A)
B)
C)

## Slide 7
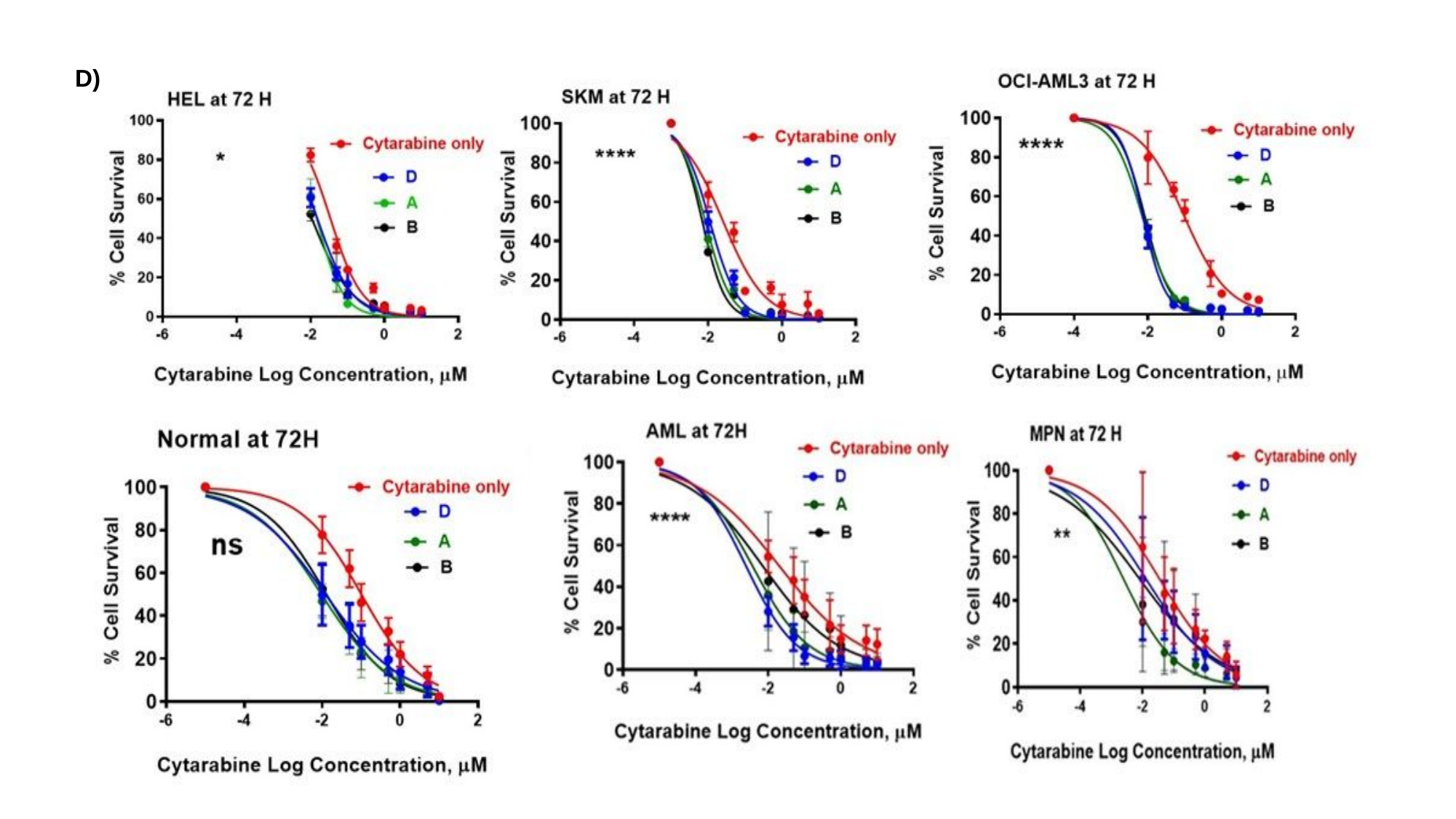

D)

## Slide 8
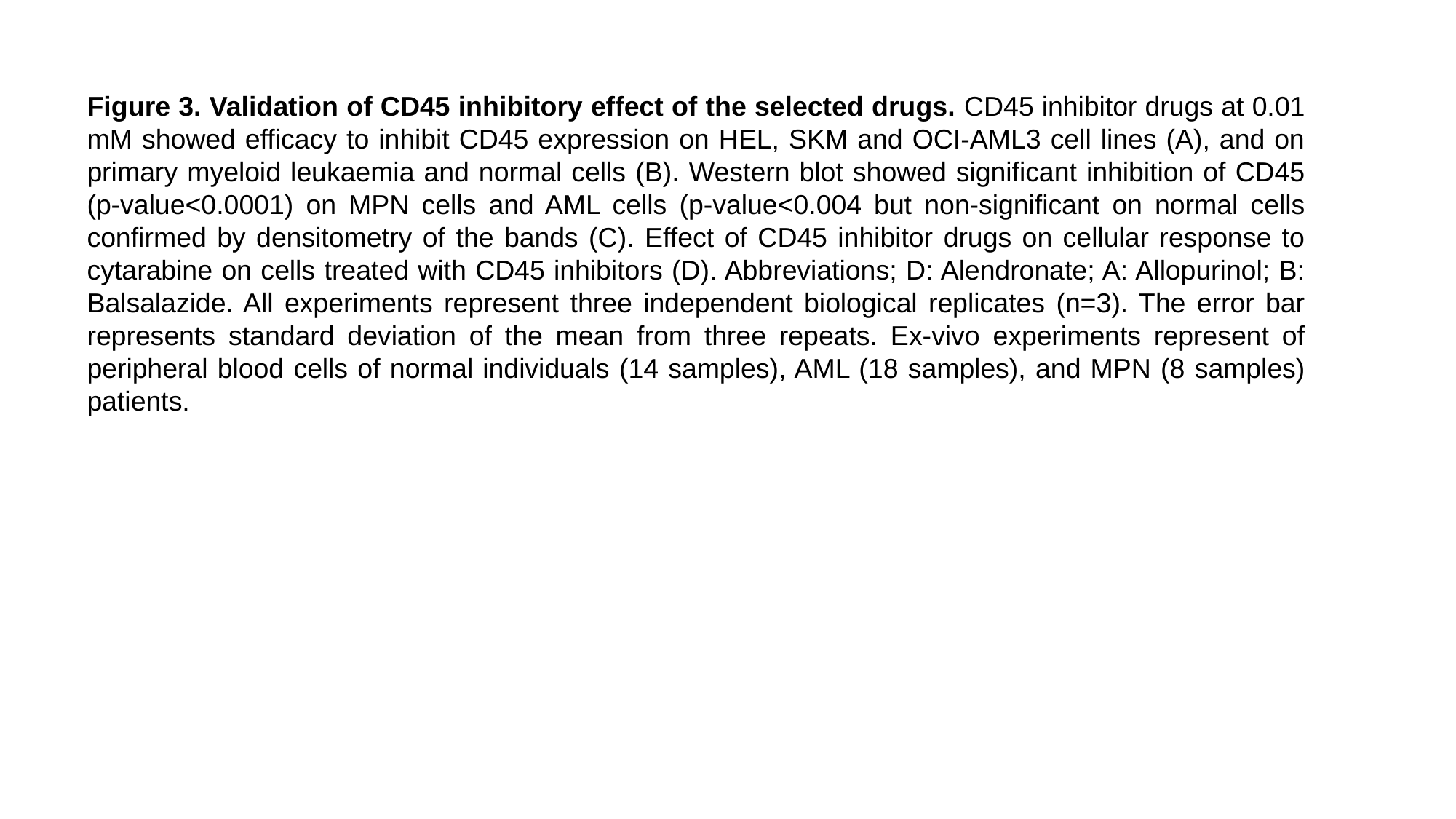

Figure 3. Validation of CD45 inhibitory effect of the selected drugs. CD45 inhibitor drugs at 0.01 mM showed efficacy to inhibit CD45 expression on HEL, SKM and OCI-AML3 cell lines (A), and on primary myeloid leukaemia and normal cells (B). Western blot showed significant inhibition of CD45 (p-value<0.0001) on MPN cells and AML cells (p-value<0.004 but non-significant on normal cells confirmed by densitometry of the bands (C). Effect of CD45 inhibitor drugs on cellular response to cytarabine on cells treated with CD45 inhibitors (D). Abbreviations; D: Alendronate; A: Allopurinol; B: Balsalazide. All experiments represent three independent biological replicates (n=3). The error bar represents standard deviation of the mean from three repeats. Ex-vivo experiments represent of peripheral blood cells of normal individuals (14 samples), AML (18 samples), and MPN (8 samples) patients.

## Slide 9
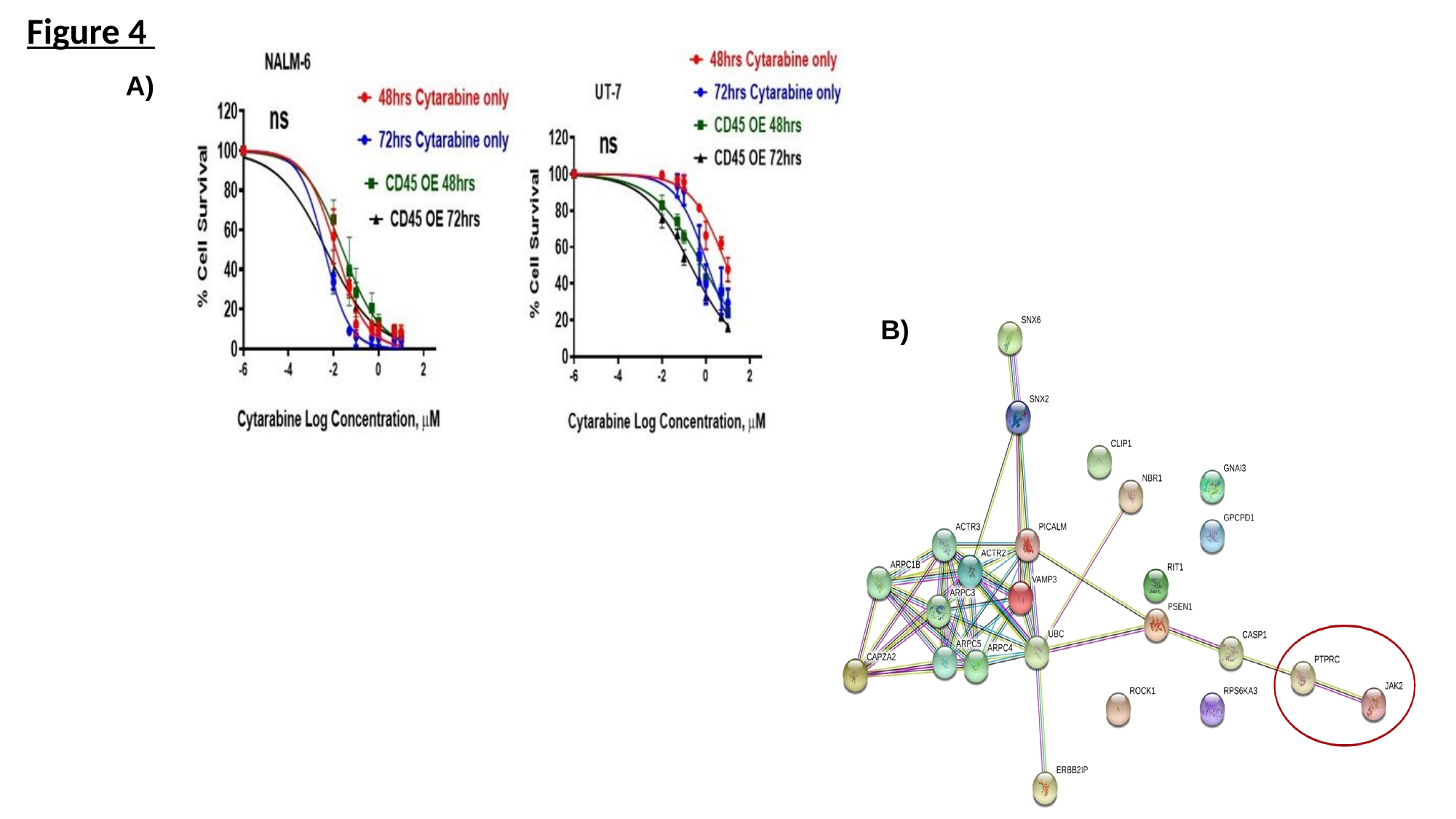

Figure 4
A)
B)

## Slide 10
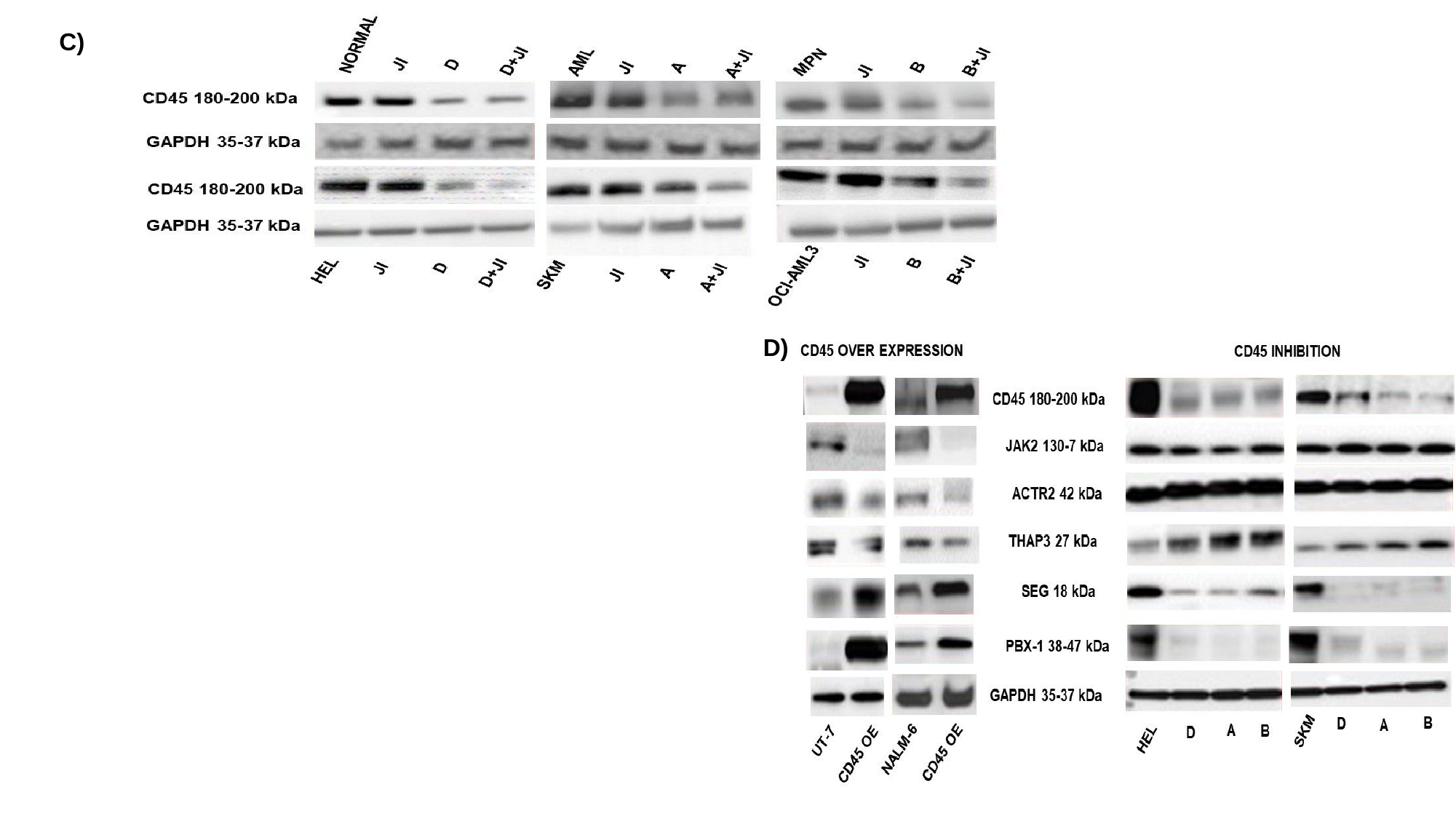

C)
D)

## Slide 11
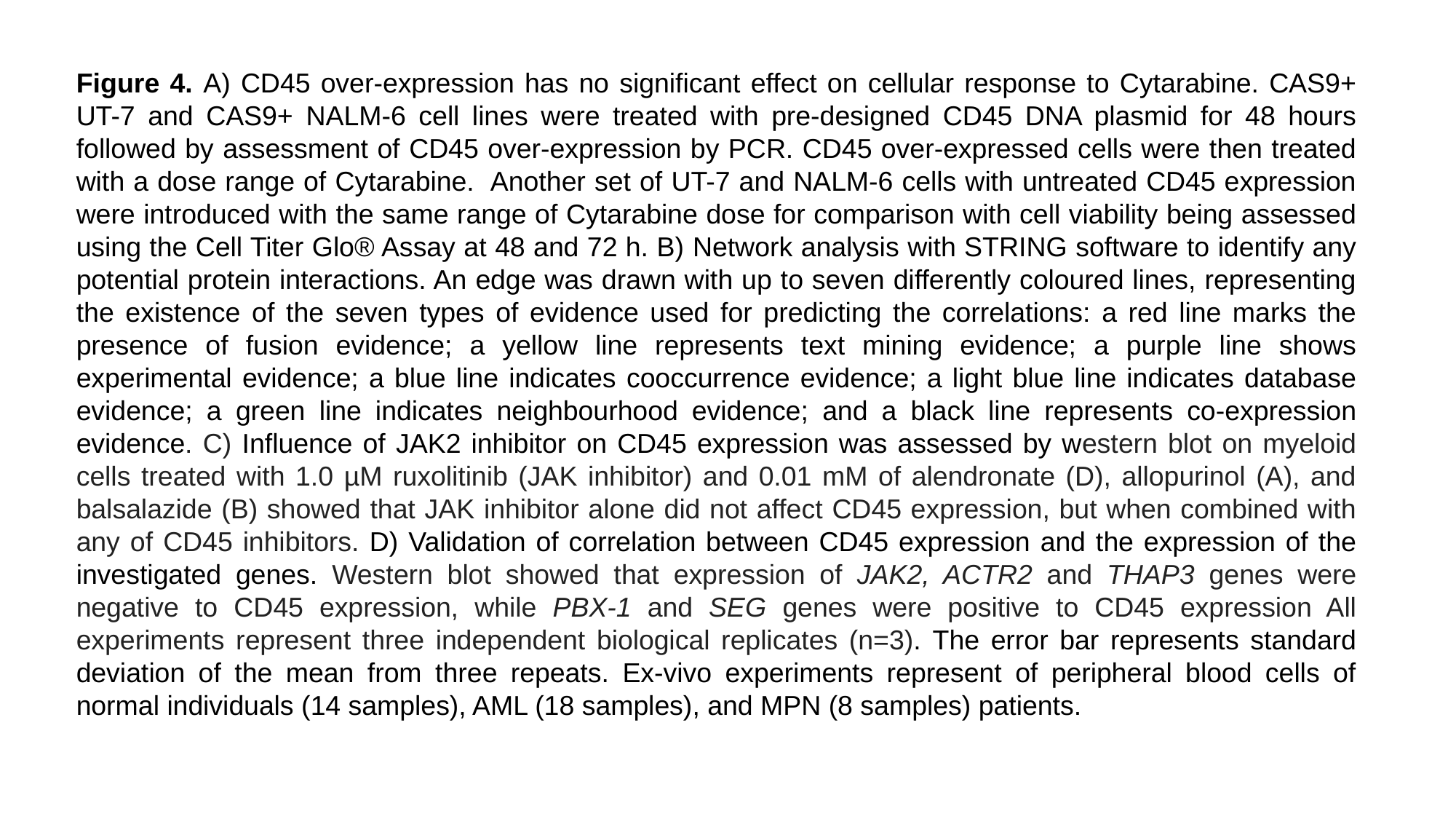

Figure 4. A) CD45 over-expression has no significant effect on cellular response to Cytarabine. CAS9+ UT-7 and CAS9+ NALM-6 cell lines were treated with pre-designed CD45 DNA plasmid for 48 hours followed by assessment of CD45 over-expression by PCR. CD45 over-expressed cells were then treated with a dose range of Cytarabine. Another set of UT-7 and NALM-6 cells with untreated CD45 expression were introduced with the same range of Cytarabine dose for comparison with cell viability being assessed using the Cell Titer Glo® Assay at 48 and 72 h. B) Network analysis with STRING software to identify any potential protein interactions. An edge was drawn with up to seven differently coloured lines, representing the existence of the seven types of evidence used for predicting the correlations: a red line marks the presence of fusion evidence; a yellow line represents text mining evidence; a purple line shows experimental evidence; a blue line indicates cooccurrence evidence; a light blue line indicates database evidence; a green line indicates neighbourhood evidence; and a black line represents co-expression evidence. C) Influence of JAK2 inhibitor on CD45 expression was assessed by western blot on myeloid cells treated with 1.0 µM ruxolitinib (JAK inhibitor) and 0.01 mM of alendronate (D), allopurinol (A), and balsalazide (B) showed that JAK inhibitor alone did not affect CD45 expression, but when combined with any of CD45 inhibitors. D) Validation of correlation between CD45 expression and the expression of the investigated genes. Western blot showed that expression of JAK2, ACTR2 and THAP3 genes were negative to CD45 expression, while PBX-1 and SEG genes were positive to CD45 expression All experiments represent three independent biological replicates (n=3). The error bar represents standard deviation of the mean from three repeats. Ex-vivo experiments represent of peripheral blood cells of normal individuals (14 samples), AML (18 samples), and MPN (8 samples) patients.

## Slide 12
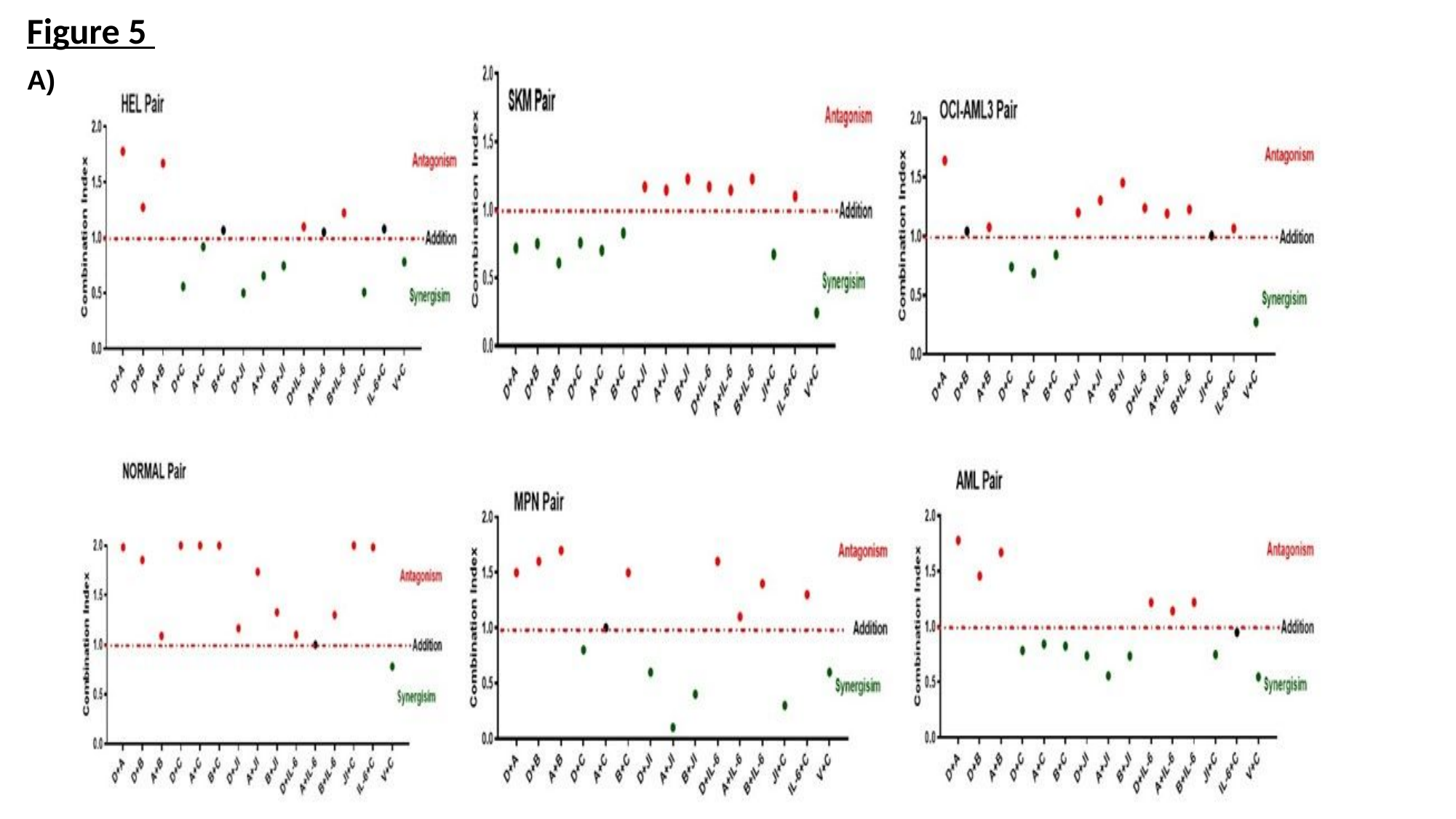

Figure 5
A)

## Slide 13
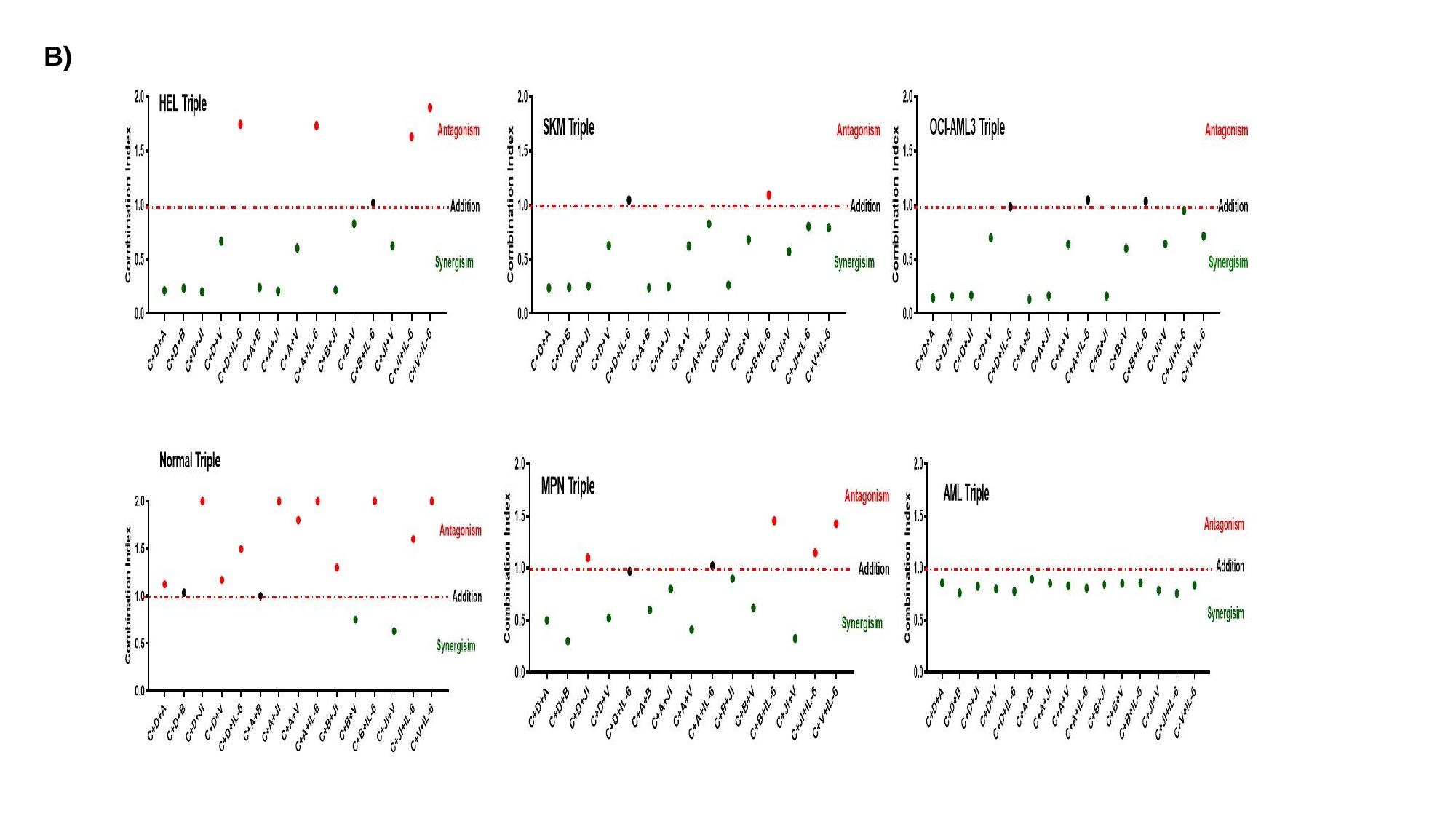

B)

## Slide 14
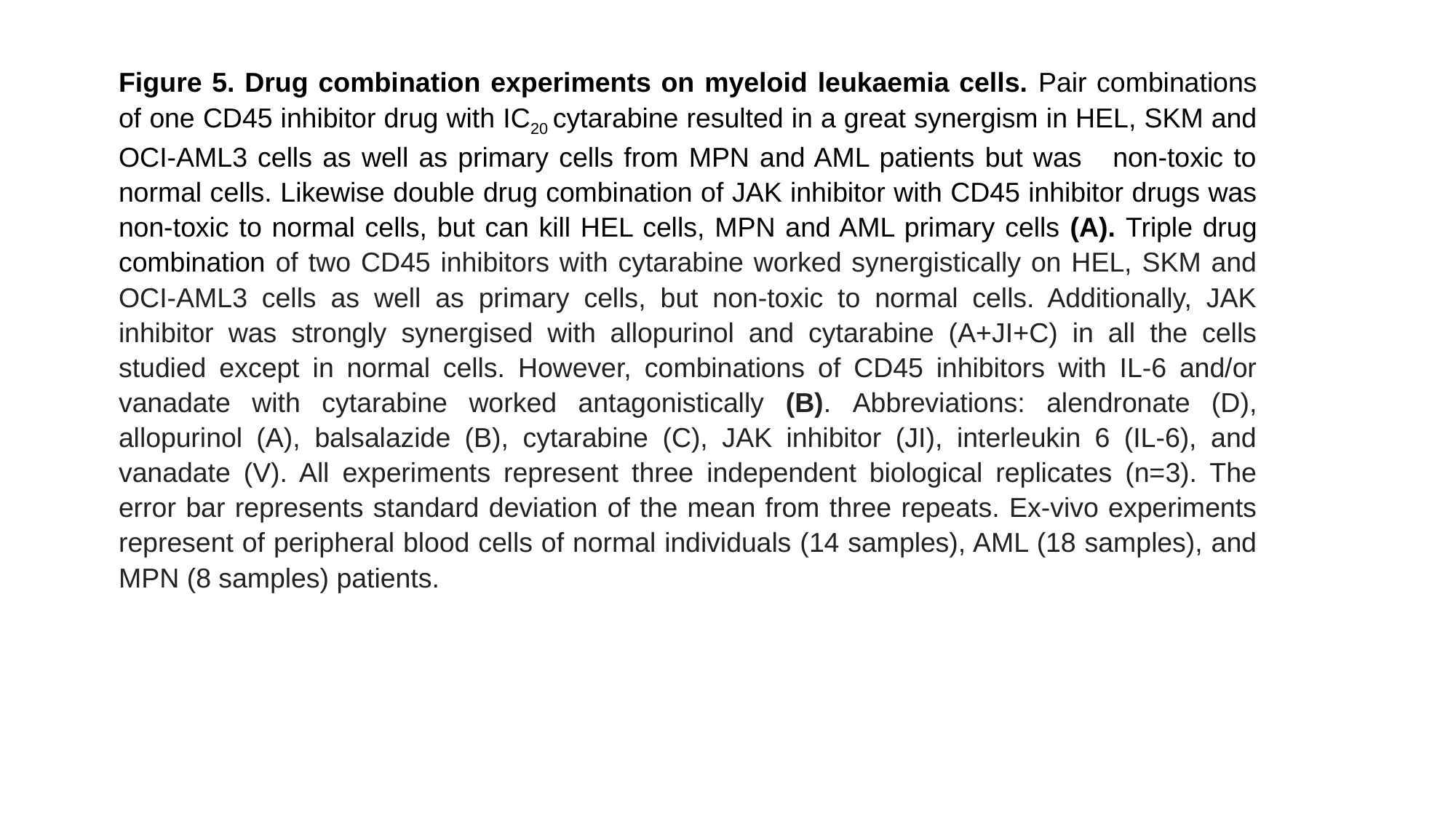

Figure 5. Drug combination experiments on myeloid leukaemia cells. Pair combinations of one CD45 inhibitor drug with IC20 cytarabine resulted in a great synergism in HEL, SKM and OCI-AML3 cells as well as primary cells from MPN and AML patients but was non-toxic to normal cells. Likewise double drug combination of JAK inhibitor with CD45 inhibitor drugs was non-toxic to normal cells, but can kill HEL cells, MPN and AML primary cells (A). Triple drug combination of two CD45 inhibitors with cytarabine worked synergistically on HEL, SKM and OCI-AML3 cells as well as primary cells, but non-toxic to normal cells. Additionally, JAK inhibitor was strongly synergised with allopurinol and cytarabine (A+JI+C) in all the cells studied except in normal cells. However, combinations of CD45 inhibitors with IL-6 and/or vanadate with cytarabine worked antagonistically (B). Abbreviations: alendronate (D), allopurinol (A), balsalazide (B), cytarabine (C), JAK inhibitor (JI), interleukin 6 (IL-6), and vanadate (V). All experiments represent three independent biological replicates (n=3). The error bar represents standard deviation of the mean from three repeats. Ex-vivo experiments represent of peripheral blood cells of normal individuals (14 samples), AML (18 samples), and MPN (8 samples) patients.

## Slide 15
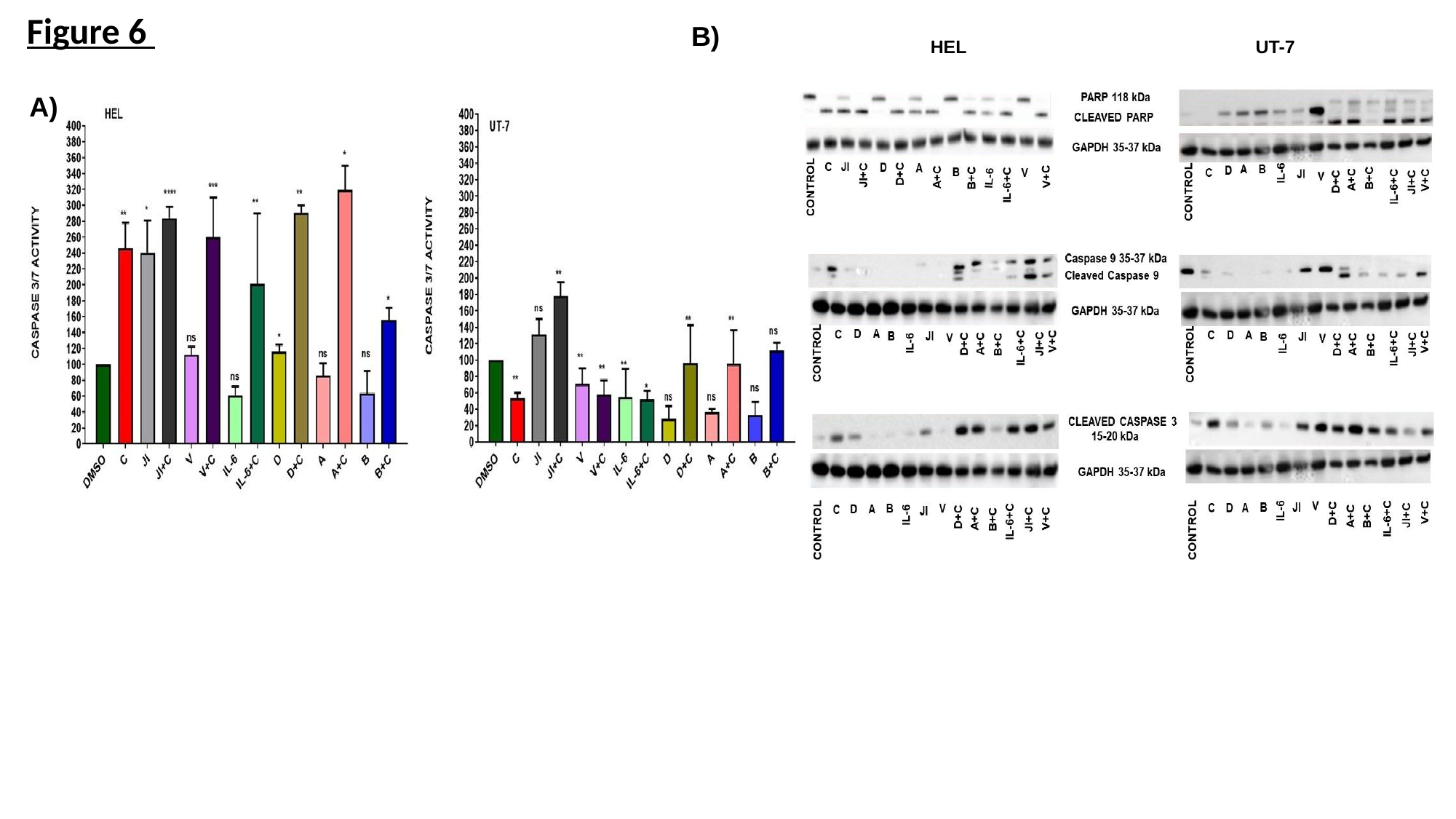

Figure 6
B)
HEL
UT-7
A)

## Slide 16
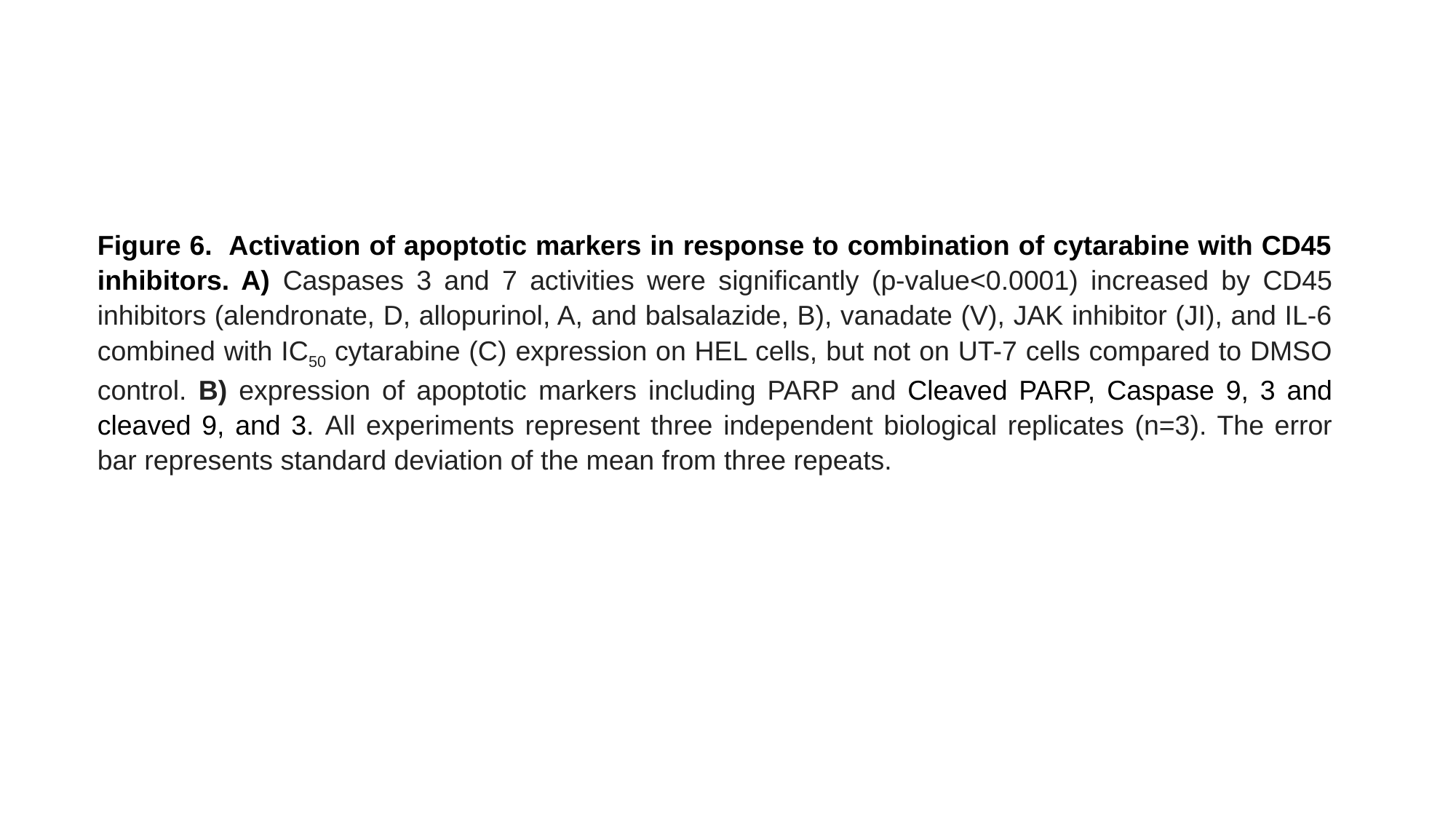

Figure 6. Activation of apoptotic markers in response to combination of cytarabine with CD45 inhibitors. A) Caspases 3 and 7 activities were significantly (p-value<0.0001) increased by CD45 inhibitors (alendronate, D, allopurinol, A, and balsalazide, B), vanadate (V), JAK inhibitor (JI), and IL-6 combined with IC50 cytarabine (C) expression on HEL cells, but not on UT-7 cells compared to DMSO control. B) expression of apoptotic markers including PARP and Cleaved PARP, Caspase 9, 3 and cleaved 9, and 3. All experiments represent three independent biological replicates (n=3). The error bar represents standard deviation of the mean from three repeats.

## Slide 17
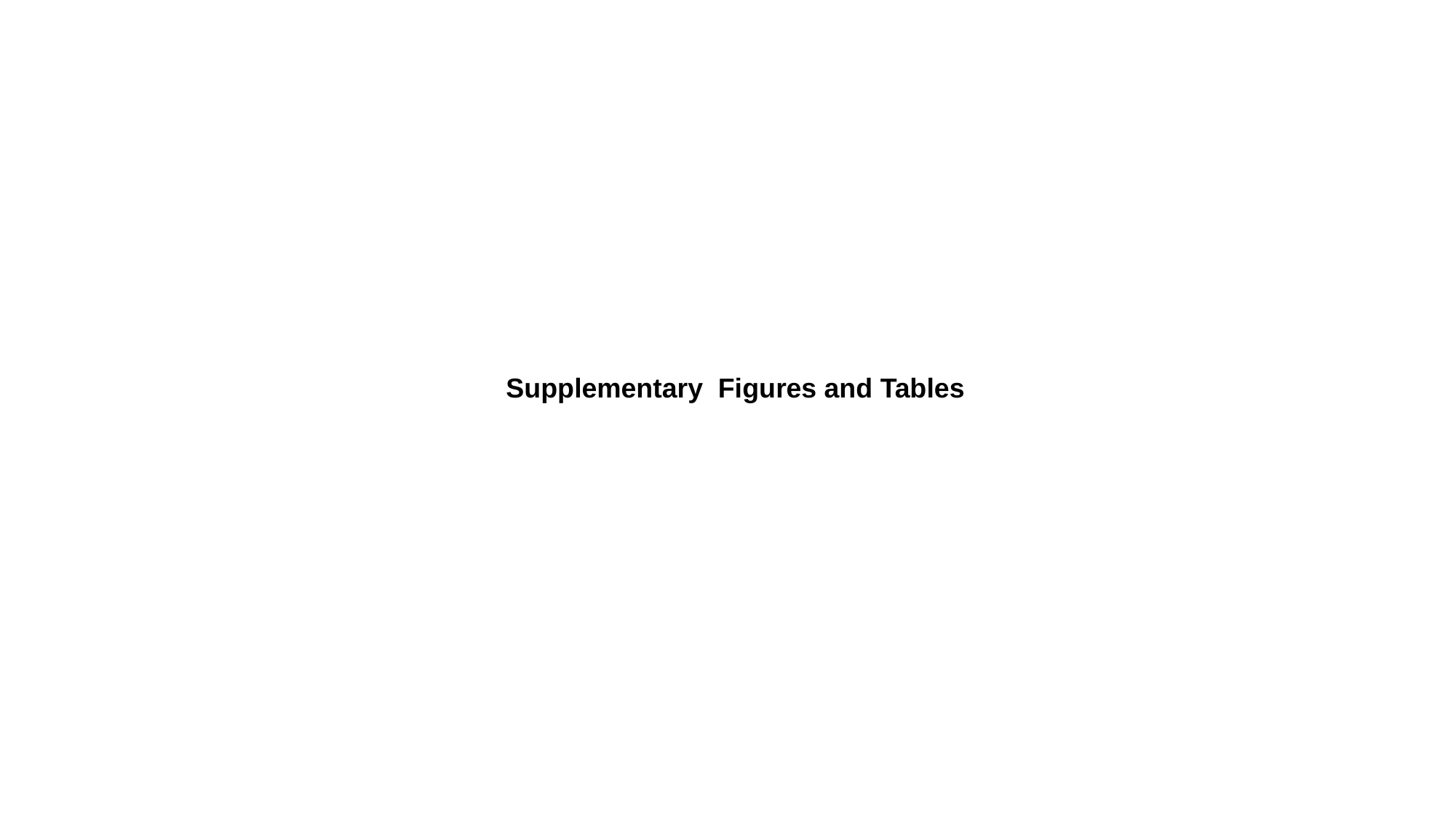

Supplementary Figures and Tables

## Slide 18
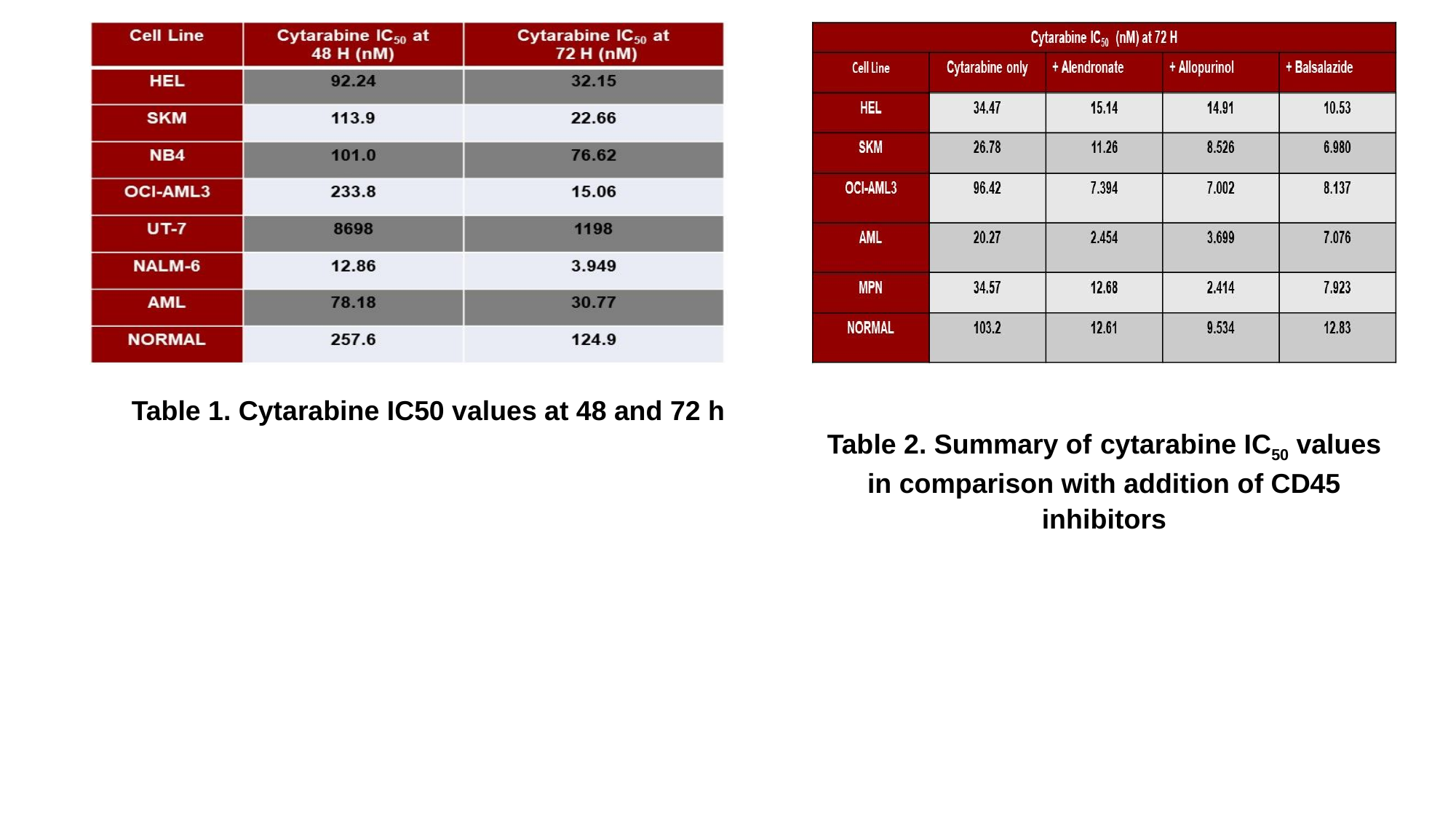

Table 1. Cytarabine IC50 values at 48 and 72 h
Table 2. Summary of cytarabine IC50 values in comparison with addition of CD45 inhibitors

## Slide 19
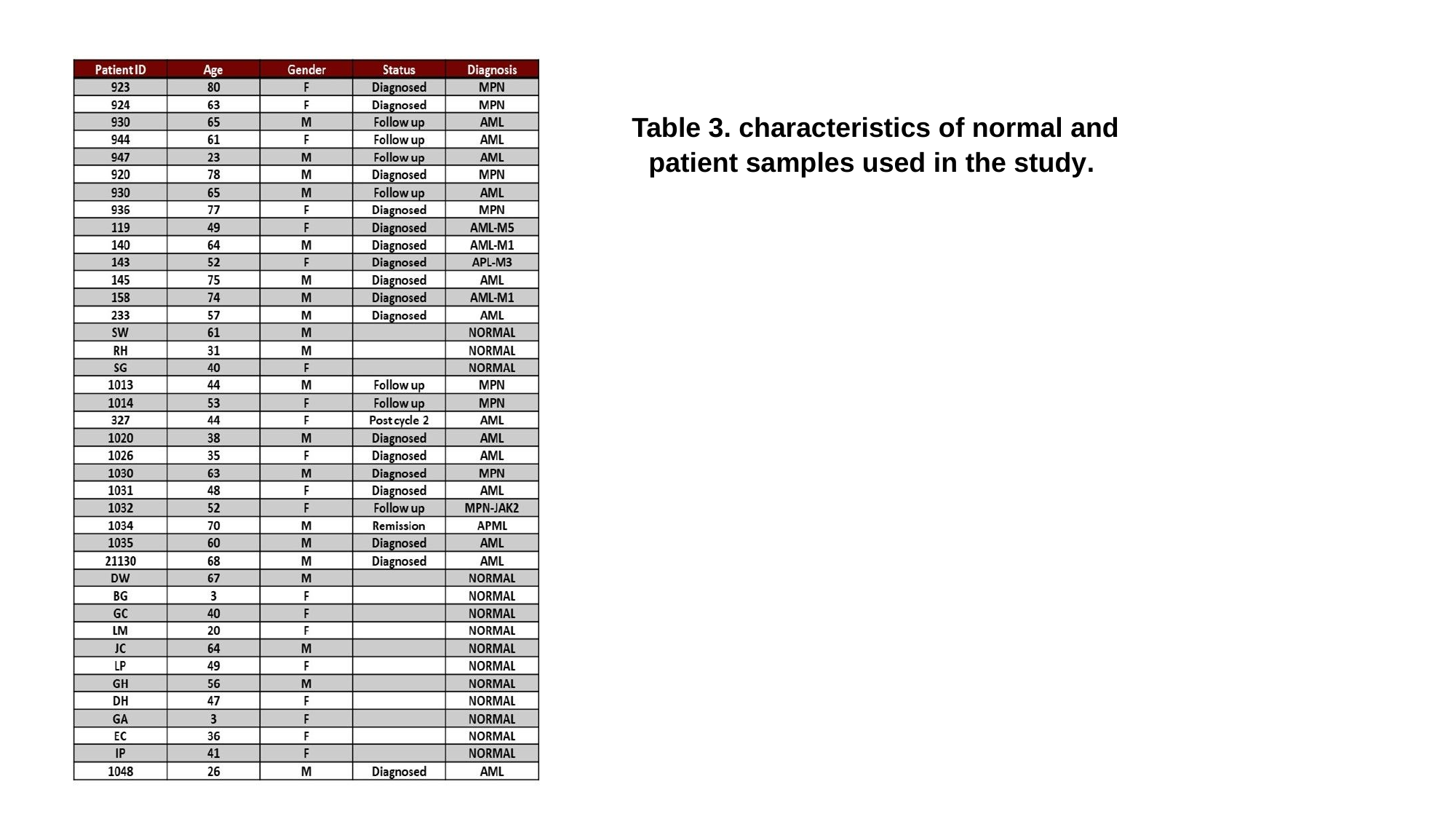

Table 3. characteristics of normal and patient samples used in the study.

## Slide 20
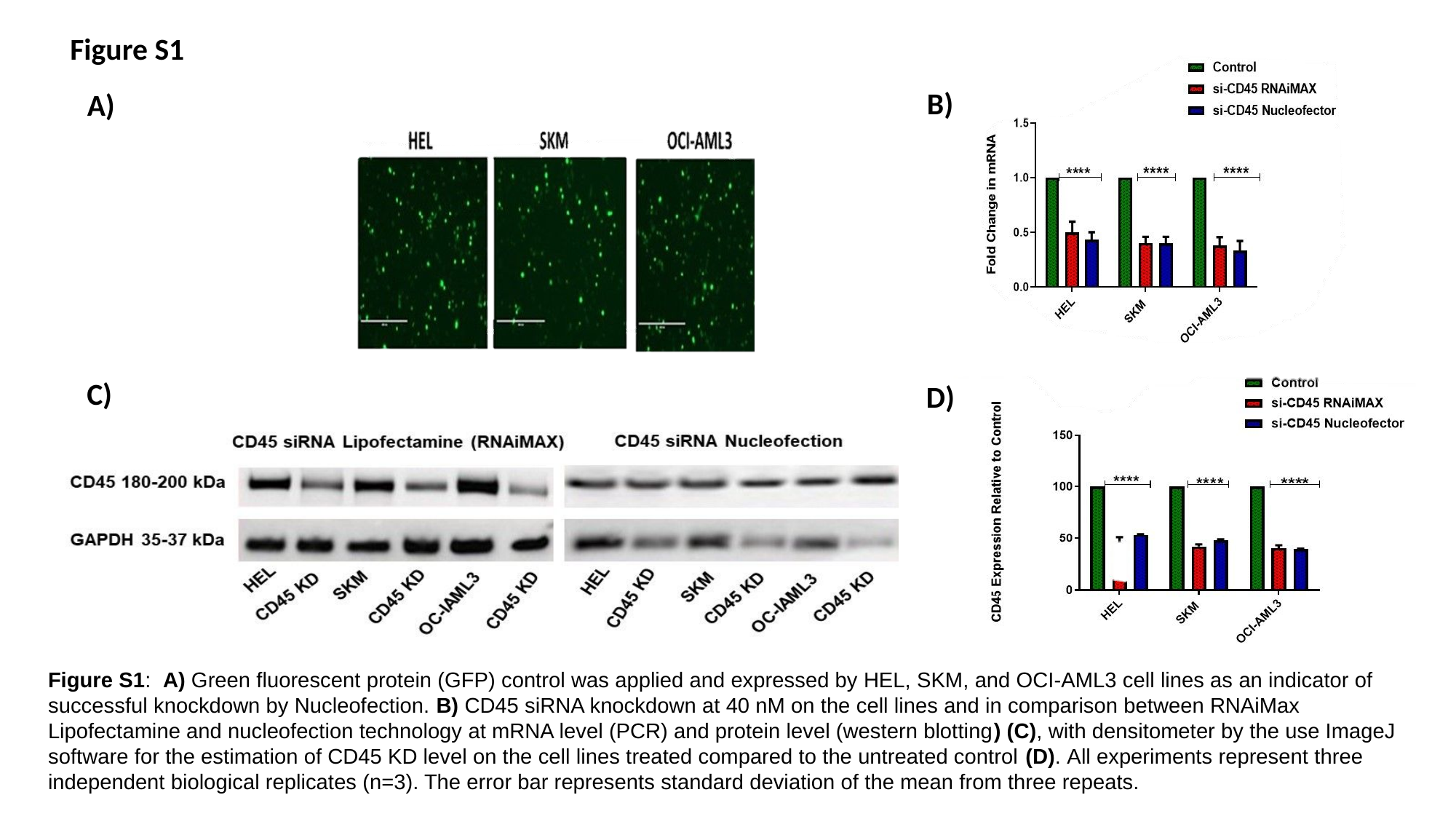

Figure S1
B)
A)
C)
D)
Figure S1: A) Green fluorescent protein (GFP) control was applied and expressed by HEL, SKM, and OCI-AML3 cell lines as an indicator of successful knockdown by Nucleofection. B) CD45 siRNA knockdown at 40 nM on the cell lines and in comparison between RNAiMax Lipofectamine and nucleofection technology at mRNA level (PCR) and protein level (western blotting) (C), with densitometer by the use ImageJ software for the estimation of CD45 KD level on the cell lines treated compared to the untreated control (D). All experiments represent three independent biological replicates (n=3). The error bar represents standard deviation of the mean from three repeats.

## Slide 21
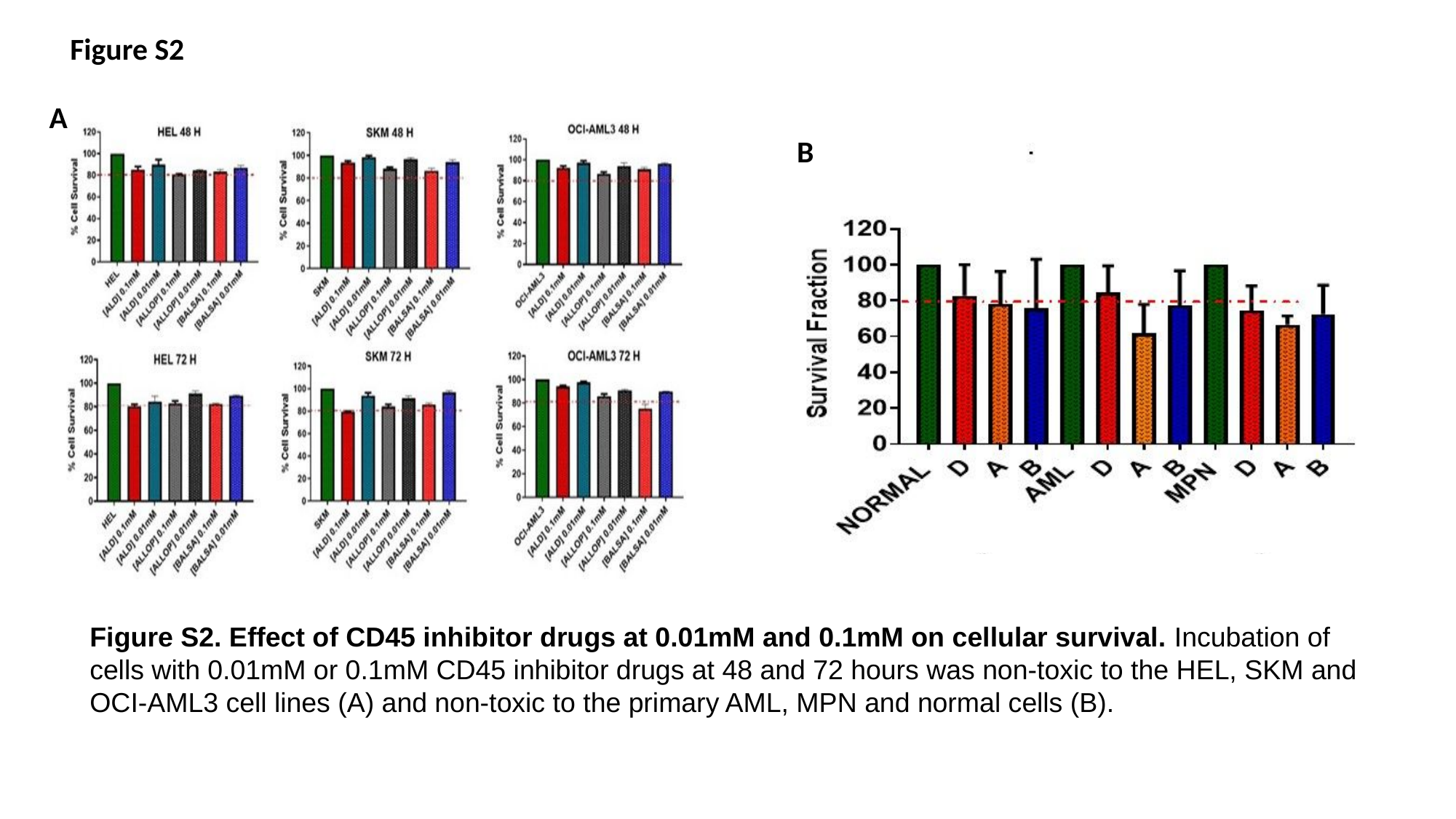

Figure S2
A
B
Figure S2. Effect of CD45 inhibitor drugs at 0.01mM and 0.1mM on cellular survival. Incubation of cells with 0.01mM or 0.1mM CD45 inhibitor drugs at 48 and 72 hours was non-toxic to the HEL, SKM and OCI-AML3 cell lines (A) and non-toxic to the primary AML, MPN and normal cells (B).

## Slide 22
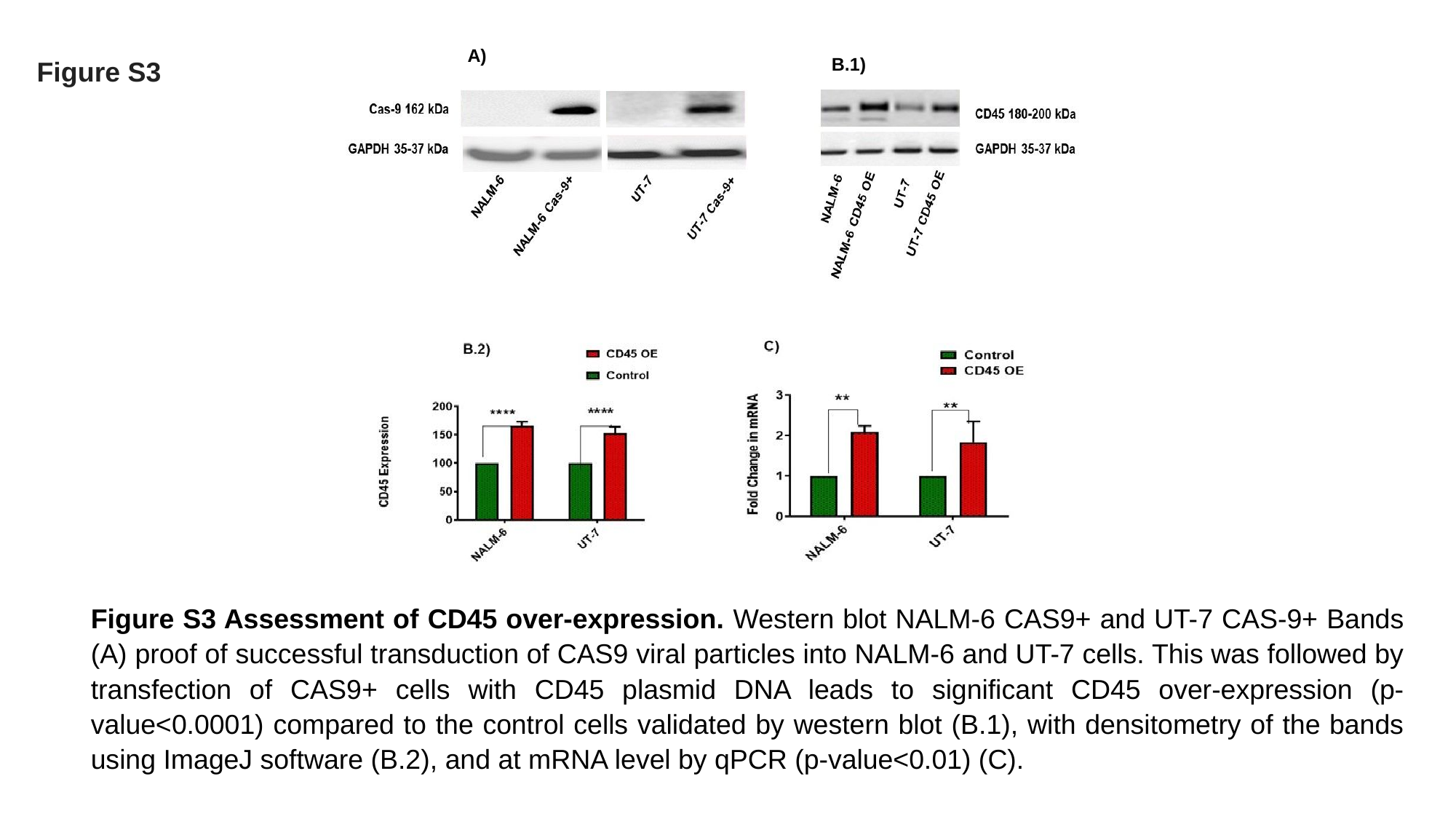

A)
B.1)
Figure S3
Figure S3 Assessment of CD45 over-expression. Western blot NALM-6 CAS9+ and UT-7 CAS-9+ Bands (A) proof of successful transduction of CAS9 viral particles into NALM-6 and UT-7 cells. This was followed by transfection of CAS9+ cells with CD45 plasmid DNA leads to significant CD45 over-expression (p-value<0.0001) compared to the control cells validated by western blot (B.1), with densitometry of the bands using ImageJ software (B.2), and at mRNA level by qPCR (p-value<0.01) (C).

## Slide 23
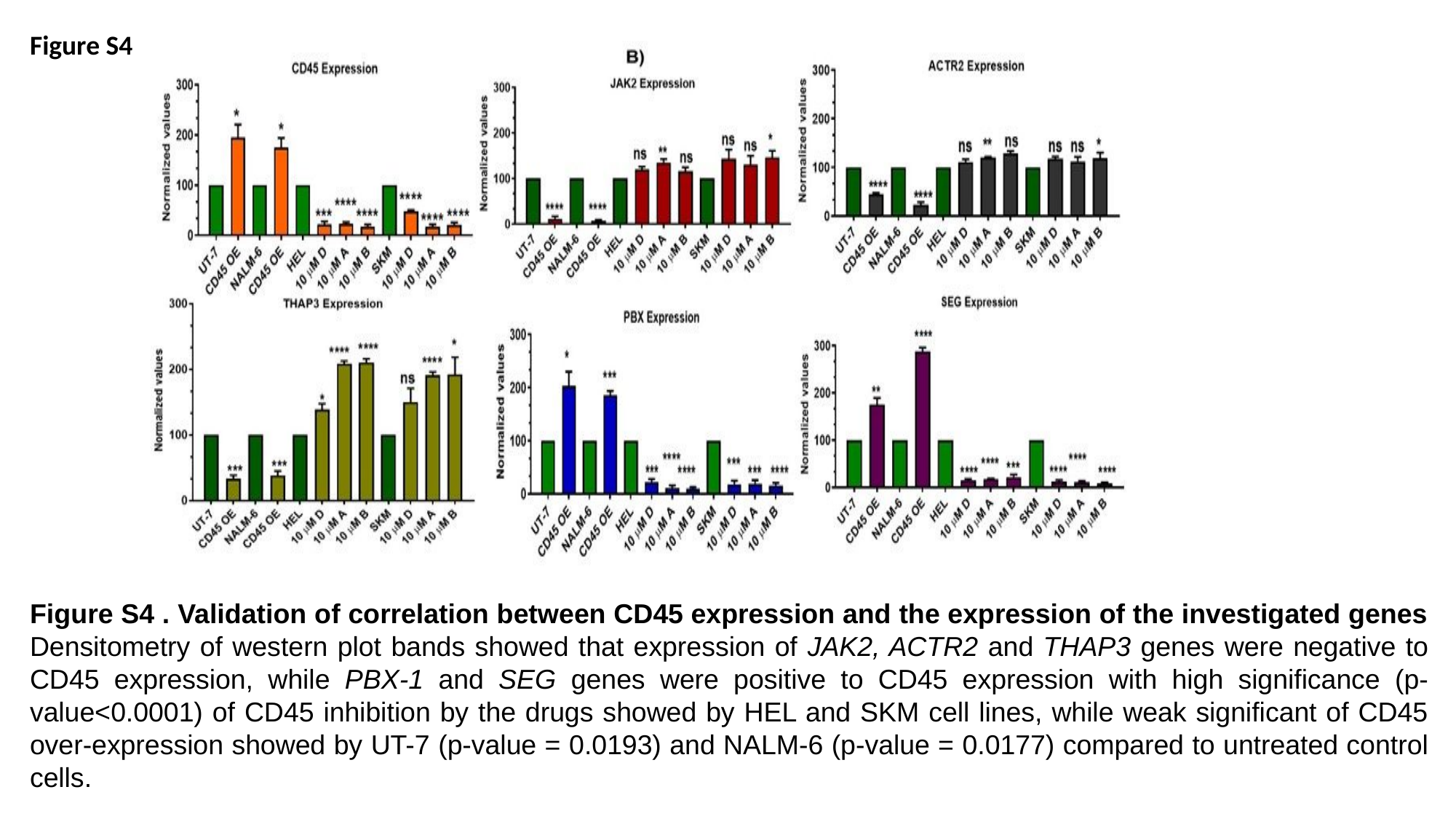

Figure S4
Figure S4 . Validation of correlation between CD45 expression and the expression of the investigated genes Densitometry of western plot bands showed that expression of JAK2, ACTR2 and THAP3 genes were negative to CD45 expression, while PBX-1 and SEG genes were positive to CD45 expression with high significance (p-value<0.0001) of CD45 inhibition by the drugs showed by HEL and SKM cell lines, while weak significant of CD45 over-expression showed by UT-7 (p-value = 0.0193) and NALM-6 (p-value = 0.0177) compared to untreated control cells.

## Slide 24
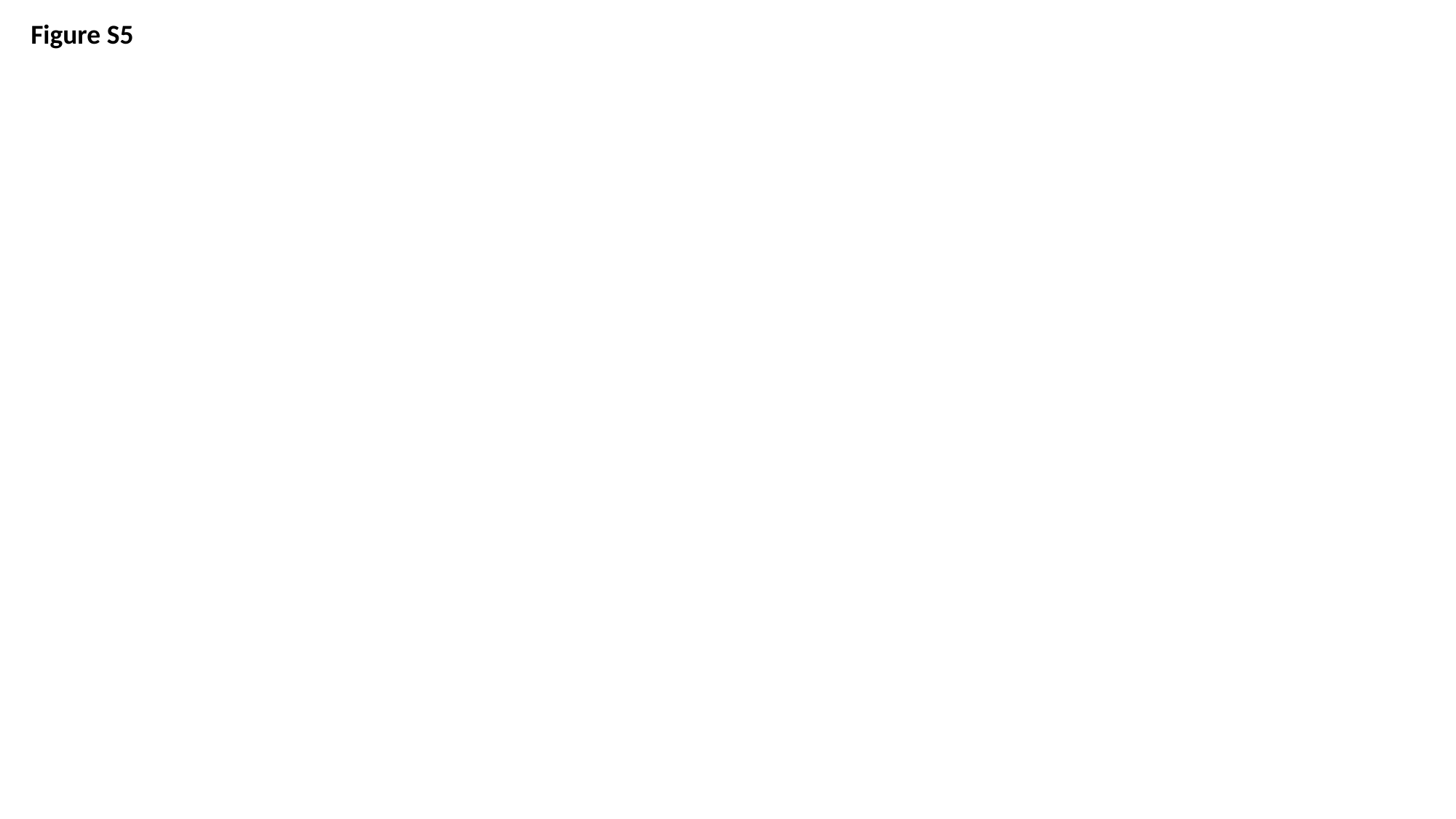

Figure S5

## Slide 25
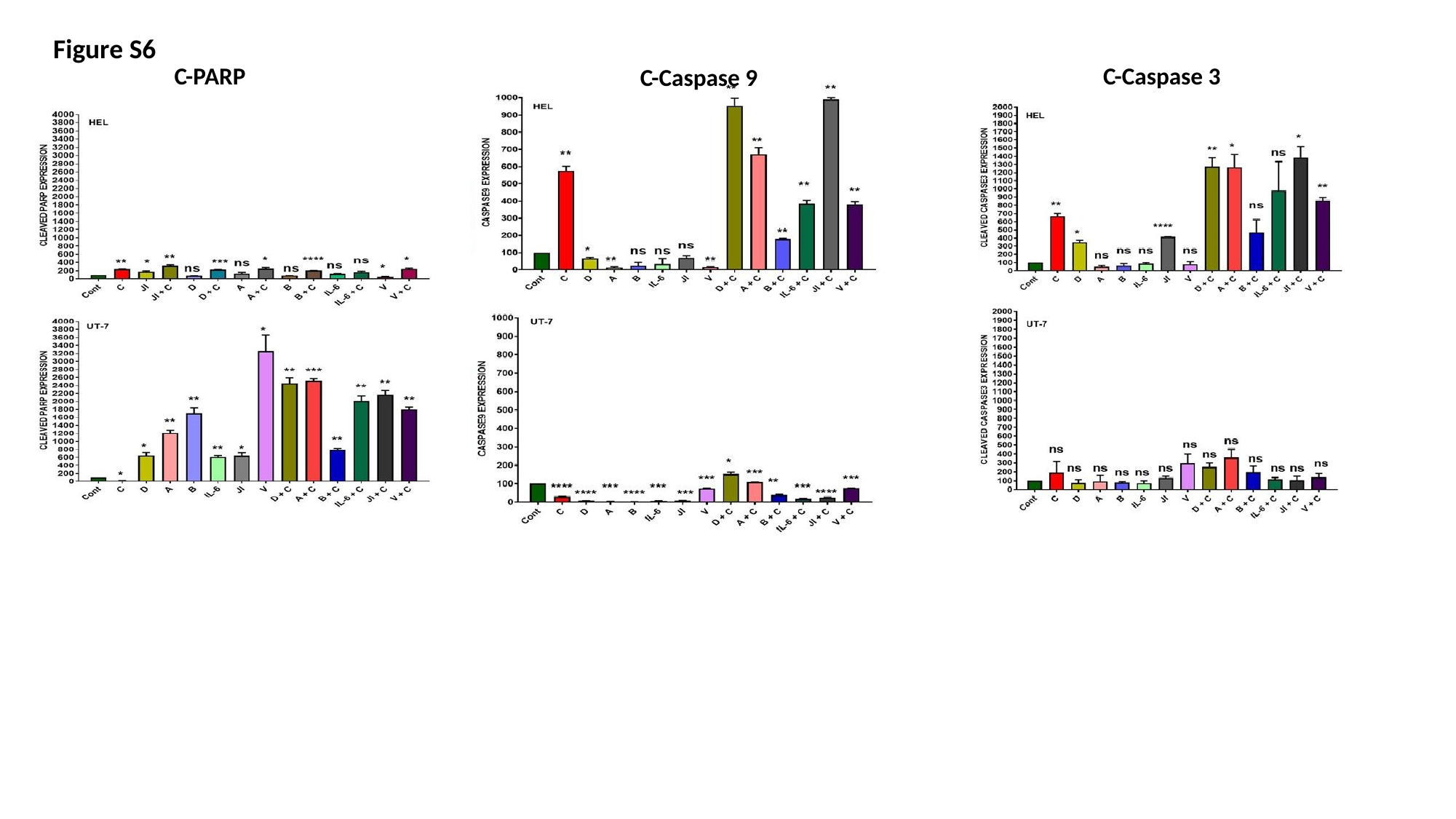

Figure S6
C-PARP
C-Caspase 3
C-Caspase 9

## Slide 26
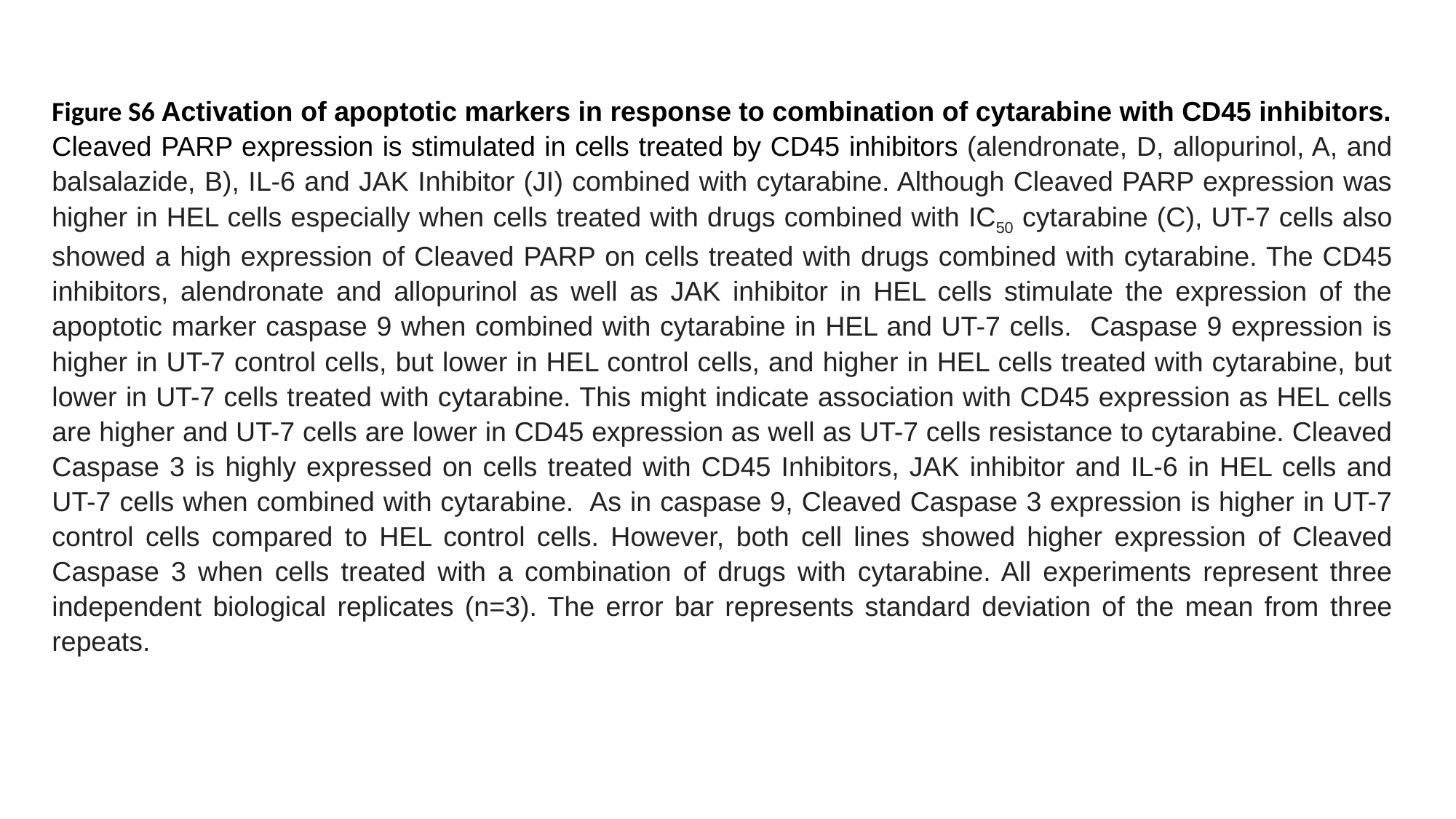

Figure S6 Activation of apoptotic markers in response to combination of cytarabine with CD45 inhibitors. Cleaved PARP expression is stimulated in cells treated by CD45 inhibitors (alendronate, D, allopurinol, A, and balsalazide, B), IL-6 and JAK Inhibitor (JI) combined with cytarabine. Although Cleaved PARP expression was higher in HEL cells especially when cells treated with drugs combined with IC50 cytarabine (C), UT-7 cells also showed a high expression of Cleaved PARP on cells treated with drugs combined with cytarabine. The CD45 inhibitors, alendronate and allopurinol as well as JAK inhibitor in HEL cells stimulate the expression of the apoptotic marker caspase 9 when combined with cytarabine in HEL and UT-7 cells. Caspase 9 expression is higher in UT-7 control cells, but lower in HEL control cells, and higher in HEL cells treated with cytarabine, but lower in UT-7 cells treated with cytarabine. This might indicate association with CD45 expression as HEL cells are higher and UT-7 cells are lower in CD45 expression as well as UT-7 cells resistance to cytarabine. Cleaved Caspase 3 is highly expressed on cells treated with CD45 Inhibitors, JAK inhibitor and IL-6 in HEL cells and UT-7 cells when combined with cytarabine. As in caspase 9, Cleaved Caspase 3 expression is higher in UT-7 control cells compared to HEL control cells. However, both cell lines showed higher expression of Cleaved Caspase 3 when cells treated with a combination of drugs with cytarabine. All experiments represent three independent biological replicates (n=3). The error bar represents standard deviation of the mean from three repeats.
